# Supplementary figures and images for: Species-specific dynamics may cause deviations from general biogeographical predictions – evidence from a population genomics study of a New Guinean endemic passerine bird family (Melampittidae)
Source: PLoS One. 2024 May 23;19(5):e0293715. doi: 10.1371/journal.pone.0293715 (PMC11115331; doi:10.1371/journal.pone.0293715)

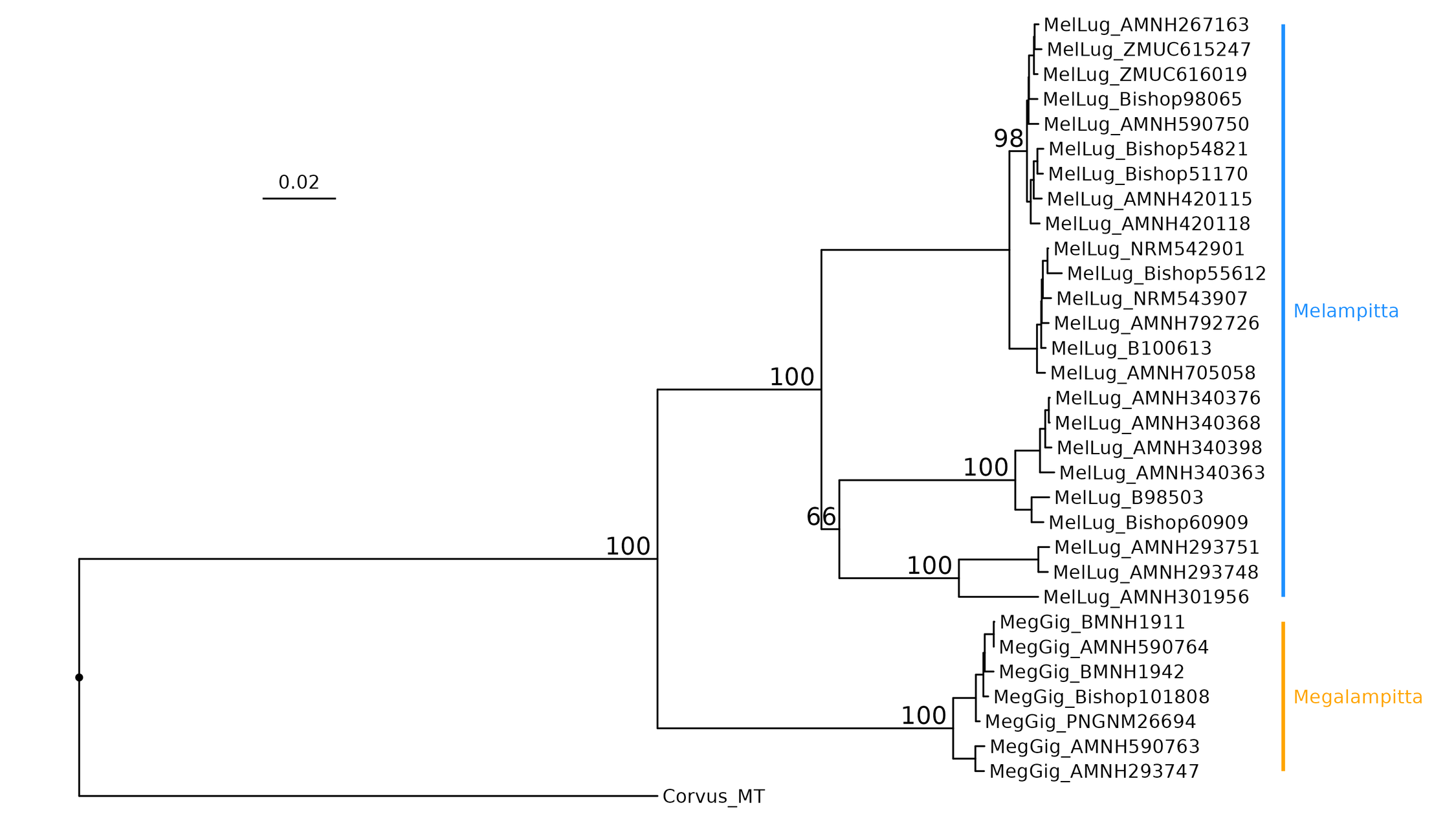

Supplement: S1 Fig — The tree was constructed using RaxML-NG applying a GTR+G substitution model. (TIF) [file pone.0293715.s001.tif]

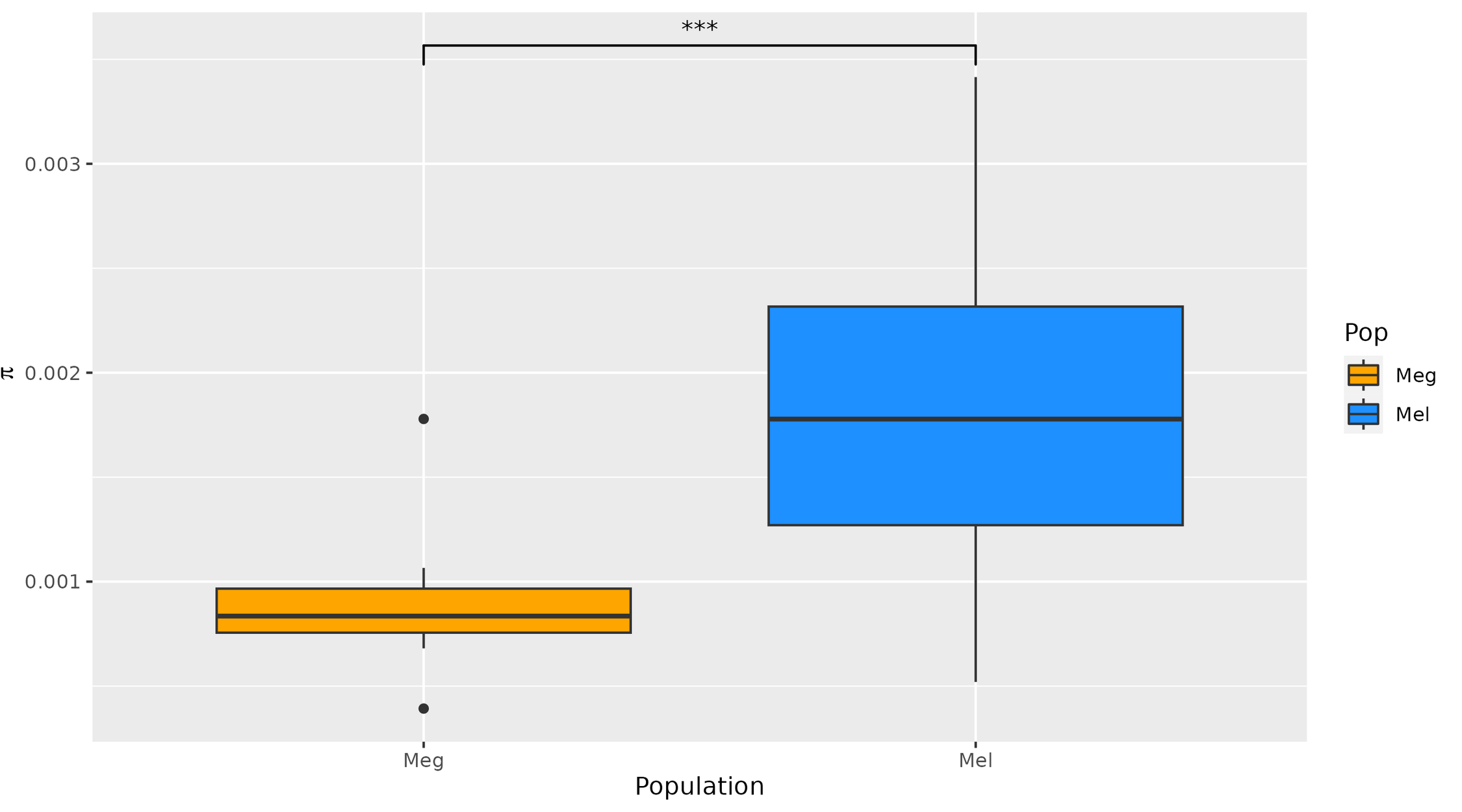

Supplement: S2 Fig — The applied statistical test was a Welch’s two sample t-test for unequal variances. (TIF) [file pone.0293715.s002.tif]

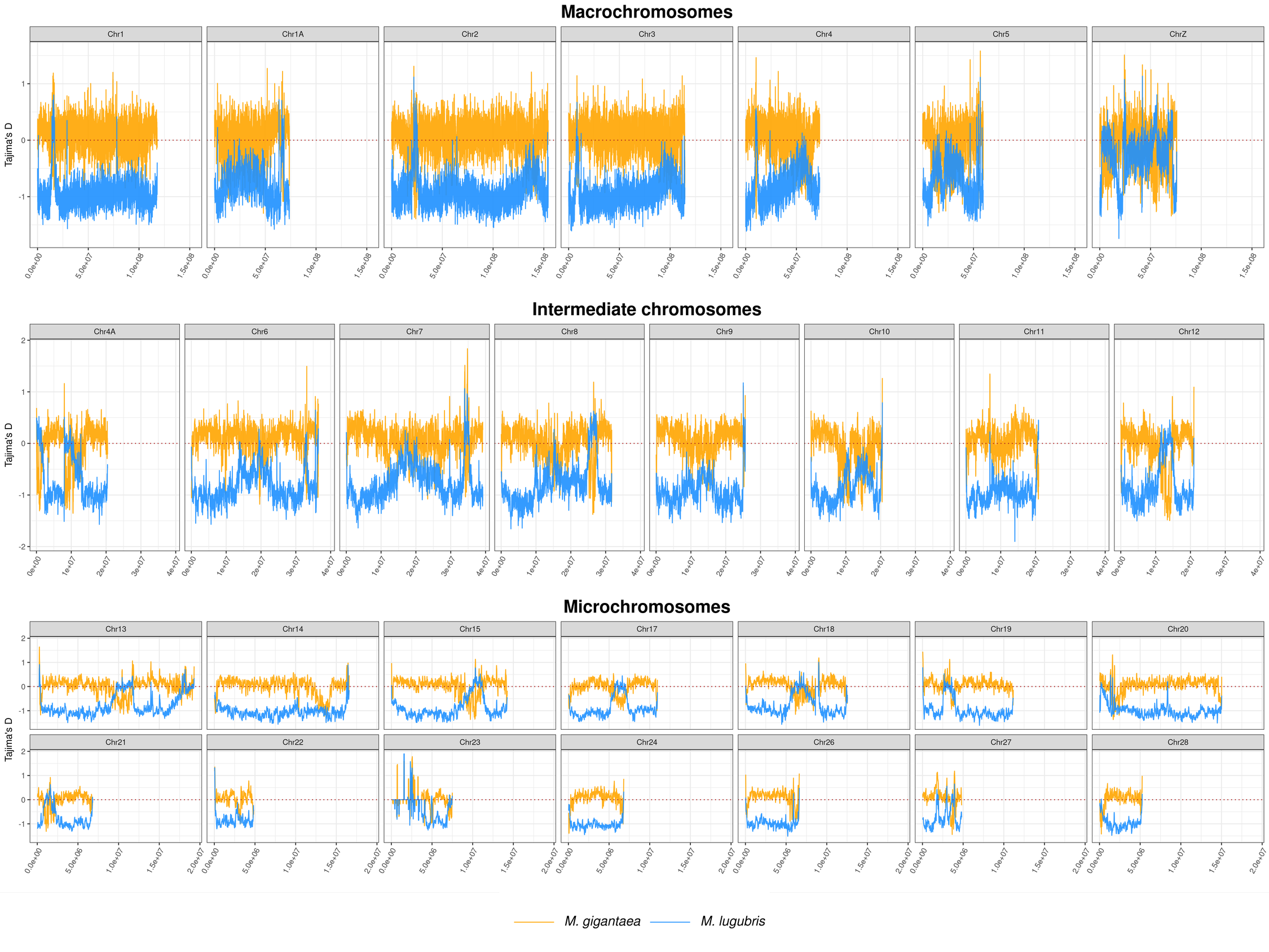

Supplement: S3 Fig — Chromosomes are divided into macrochromosomes (> = 40 Mbp), intermediate chromosomes (> = 20 Mbp, < 40 Mbp) and microchromosomes (< 20 Mbp). Values are consistently negative across most of each chromosome in M. lugubris (blue) and slightly positive in M. gigantaea (orange). (TIF) [file pone.0293715.s003.tif]

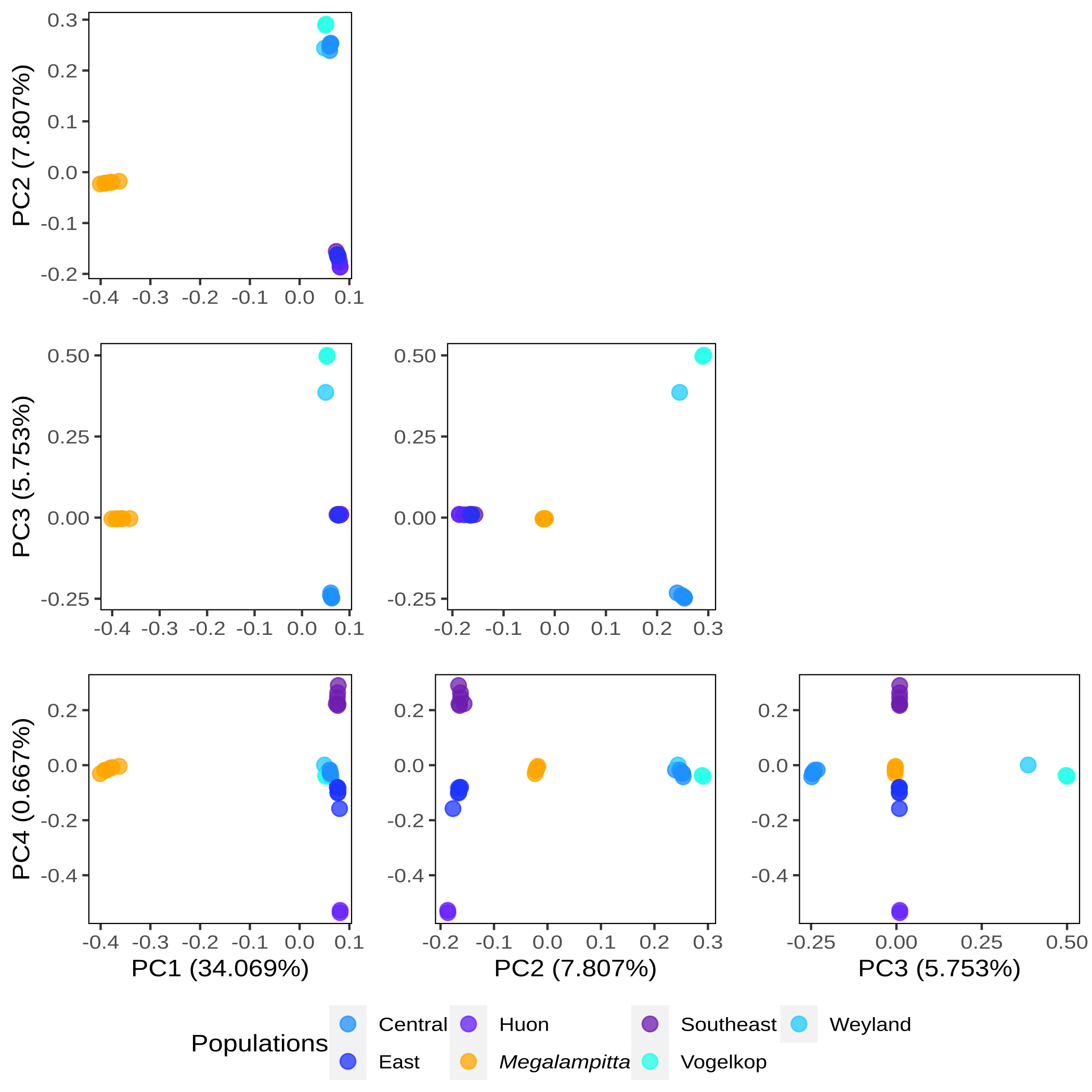

Supplement: S4 Fig — Weyland represents one individual (AMNH 301956) that was collected between our Western and Central populations and is assigned to the Western population in most other analyses. (TIF) [file pone.0293715.s004.tif]

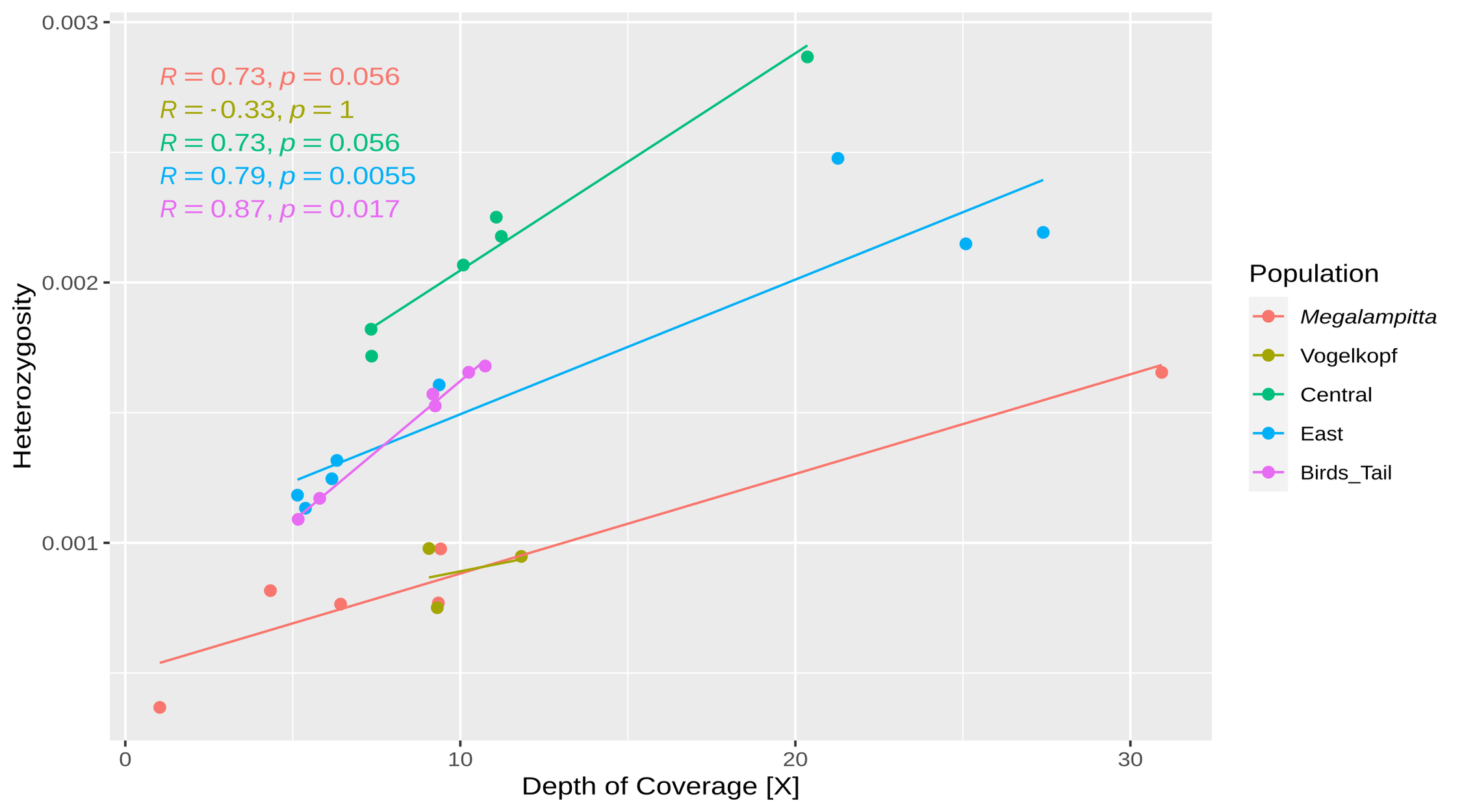

Supplement: S5 Fig — Slopes are similar between populations/species and M. gigantaea still shows lower heterozygosity than most M. lugubris populations when comparing individuals with similar DoC. To fit regression lines, we applied Kendall’s rank correlation coefficient as it is recommended for smaller sample sizes containing outliers [Kendall MG. A New Measure of Rank Correlation. Biometrika. 1938;30(1/2):81–93.]. (TIF) [file pone.0293715.s005.tif]

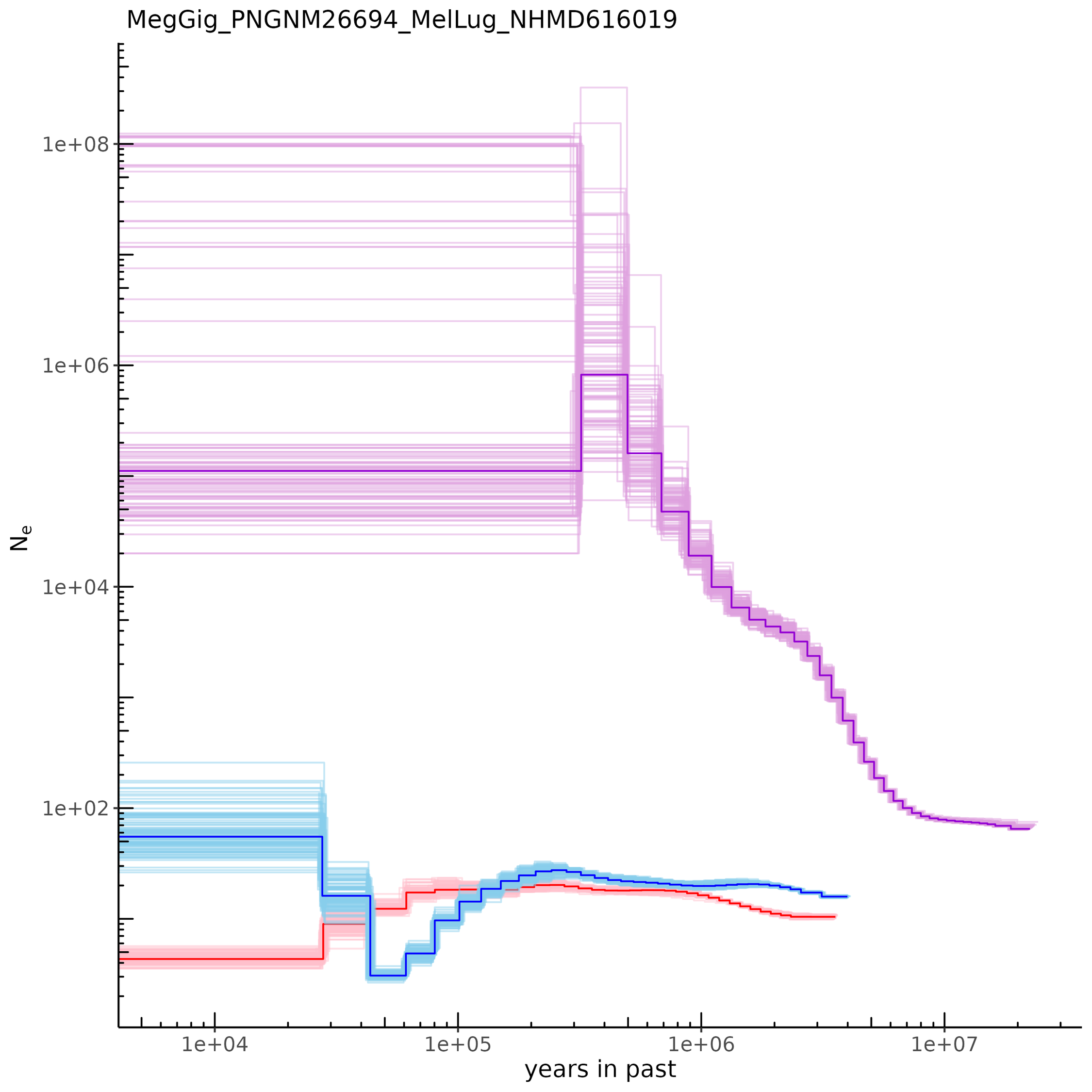

Supplement: S6 Fig — (TIF) [file pone.0293715.s006.tif]

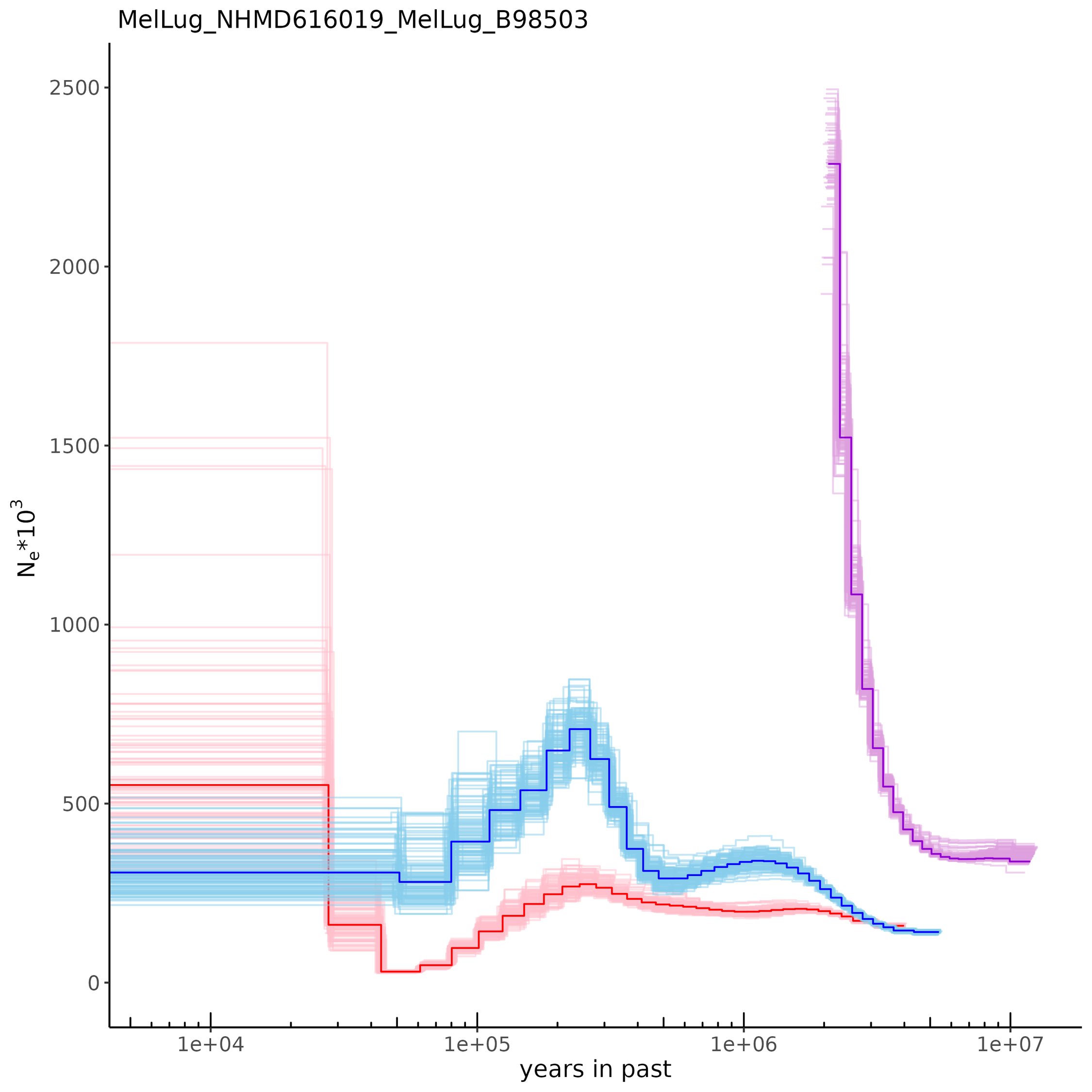

Supplement: S7 Fig — (TIF) [file pone.0293715.s007.tif]

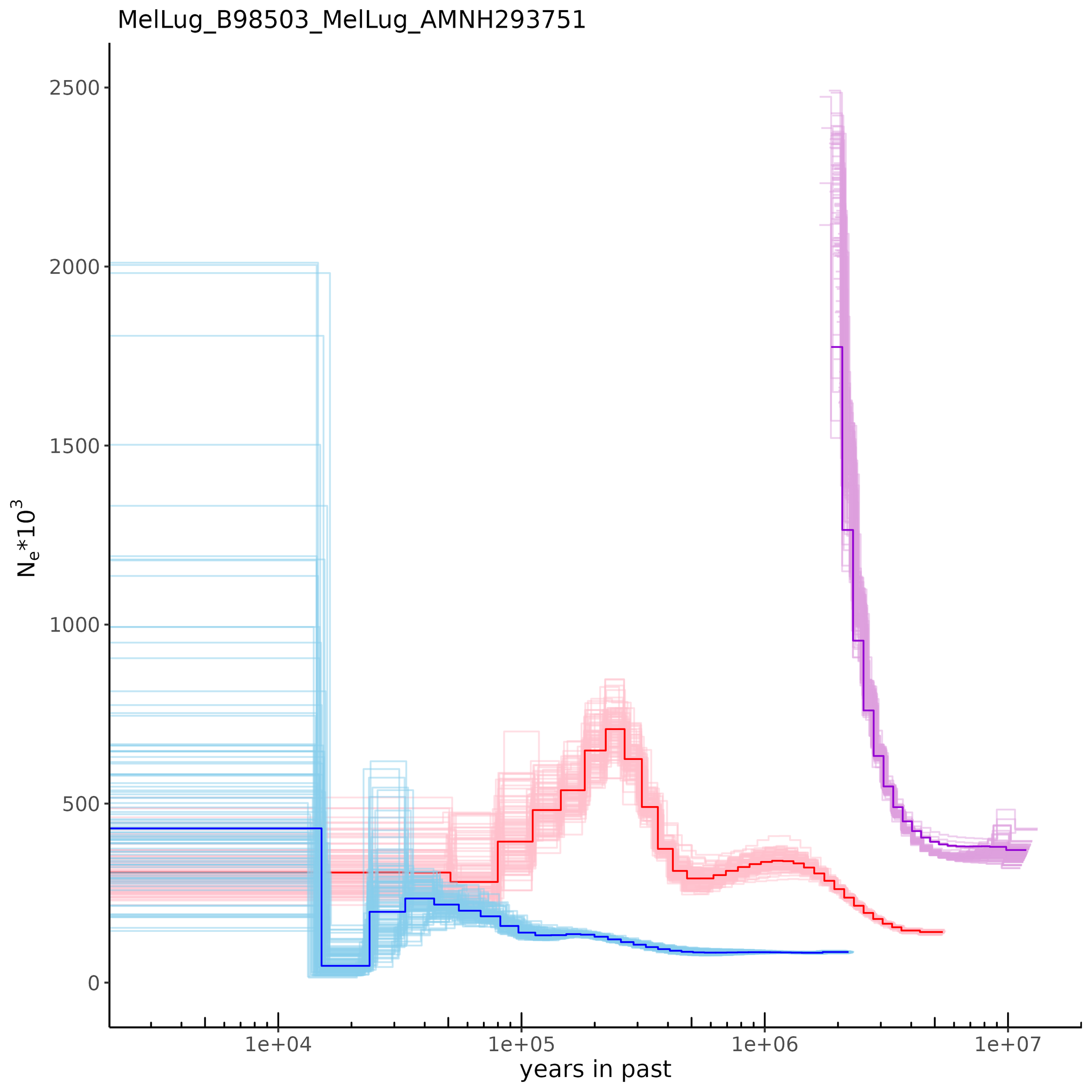

Supplement: S8 Fig — (TIF) [file pone.0293715.s008.tif]

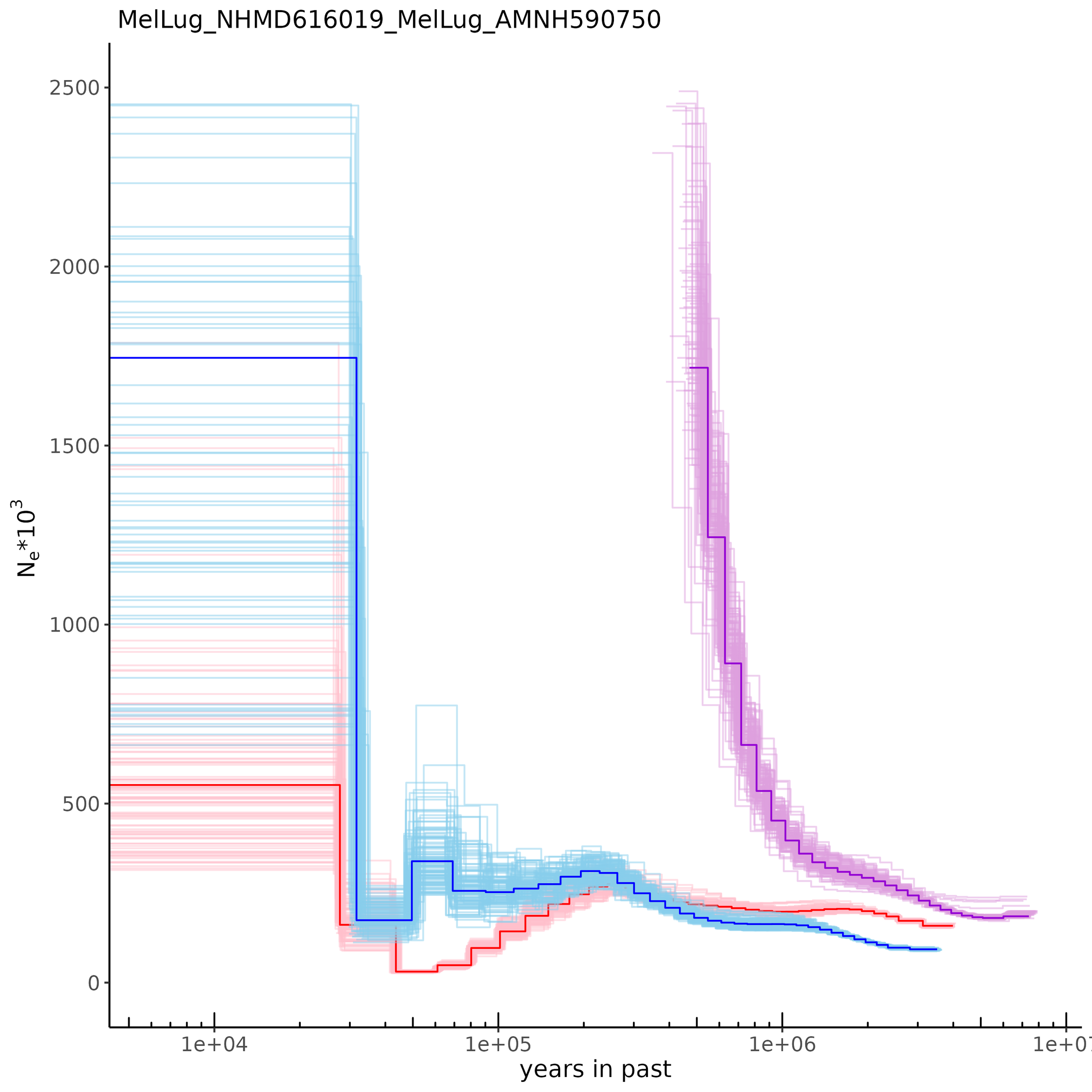

Supplement: S9 Fig — (TIF) [file pone.0293715.s009.tif]

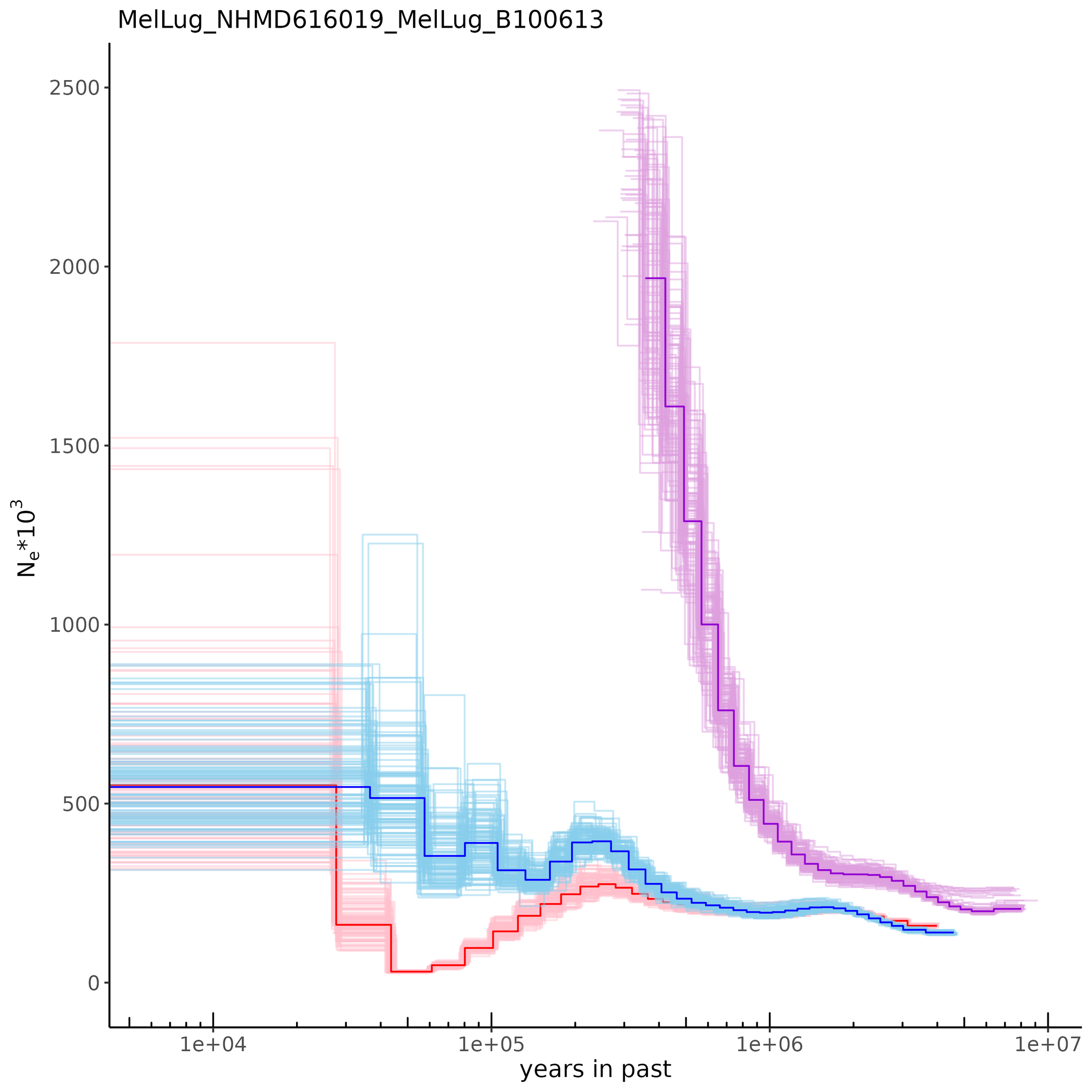

Supplement: S10 Fig — (TIF) [file pone.0293715.s010.tif]

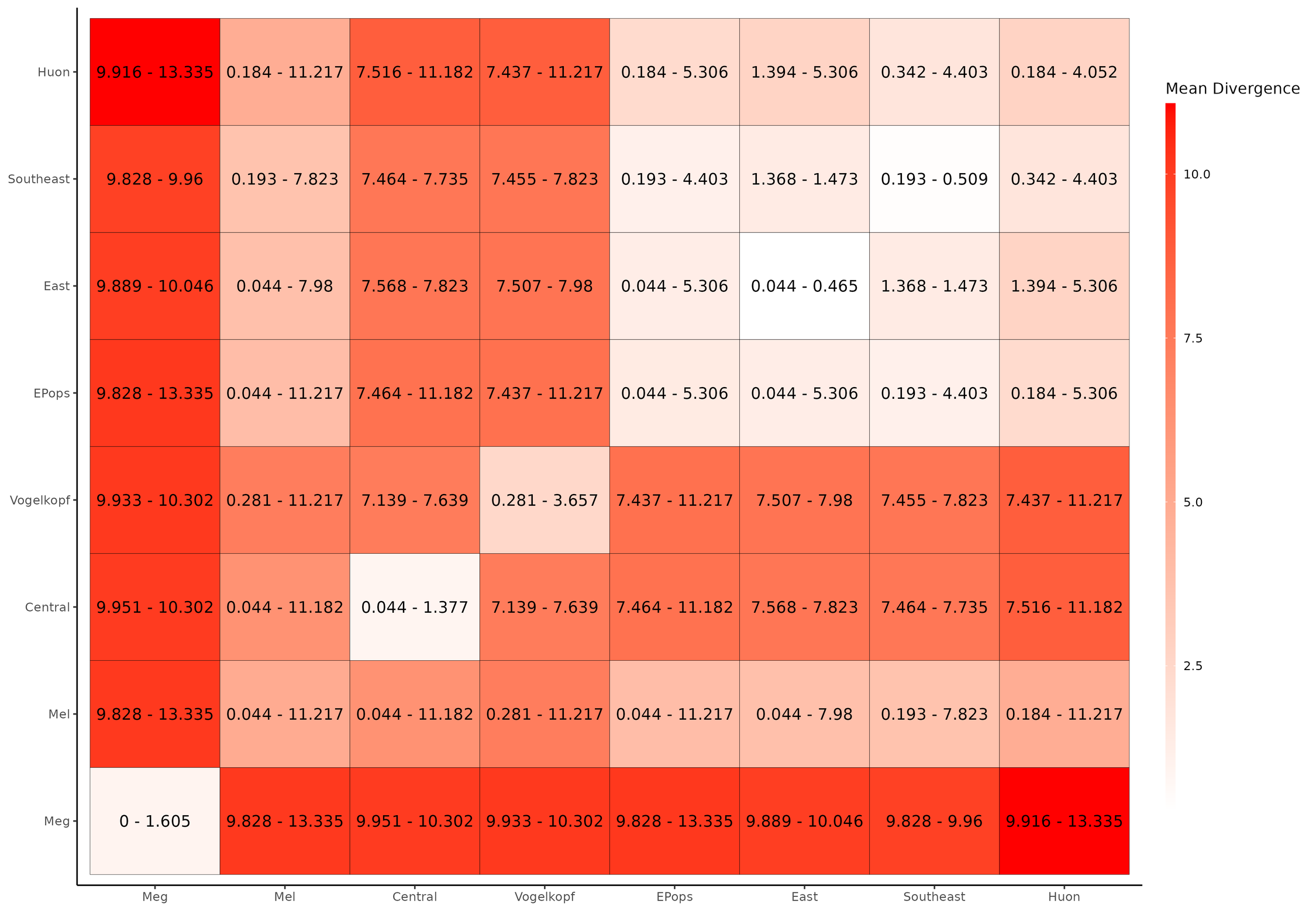

Supplement: S11 Fig — (TIF) [file pone.0293715.s011.tif]

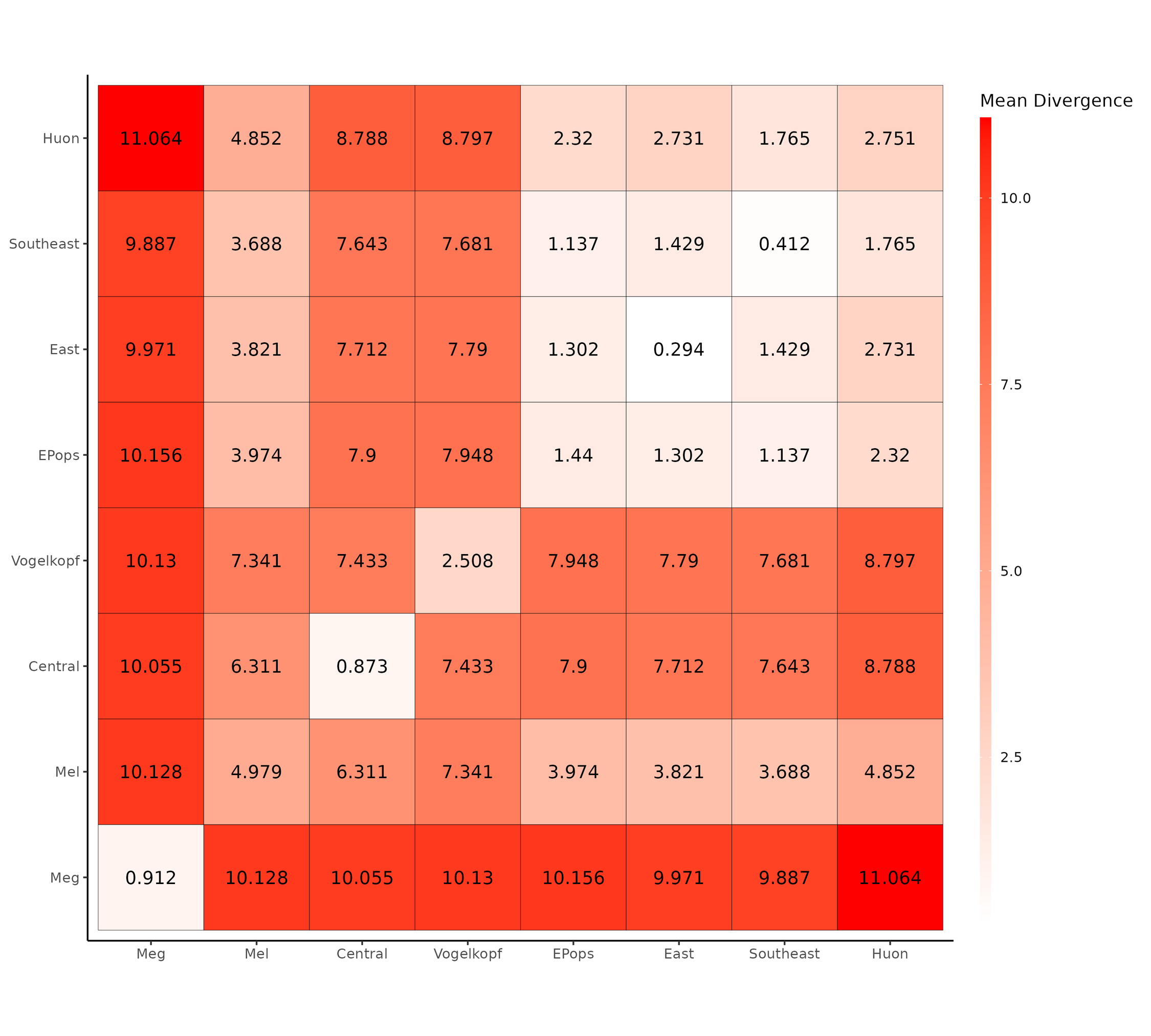

Supplement: S12 Fig — (TIF) [file pone.0293715.s012.tif]

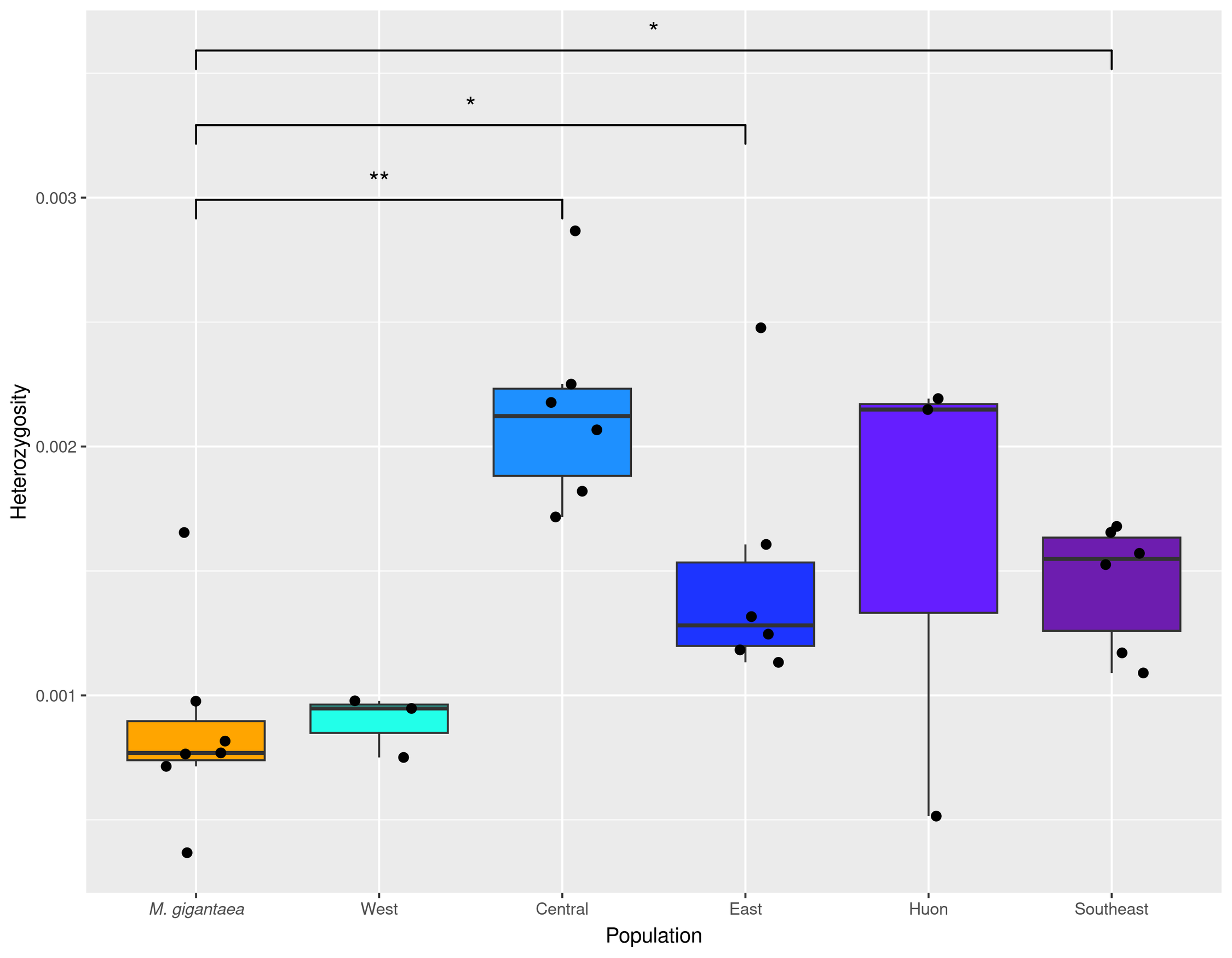

Supplement: S13 Fig — (TIF) [file pone.0293715.s013.tif]

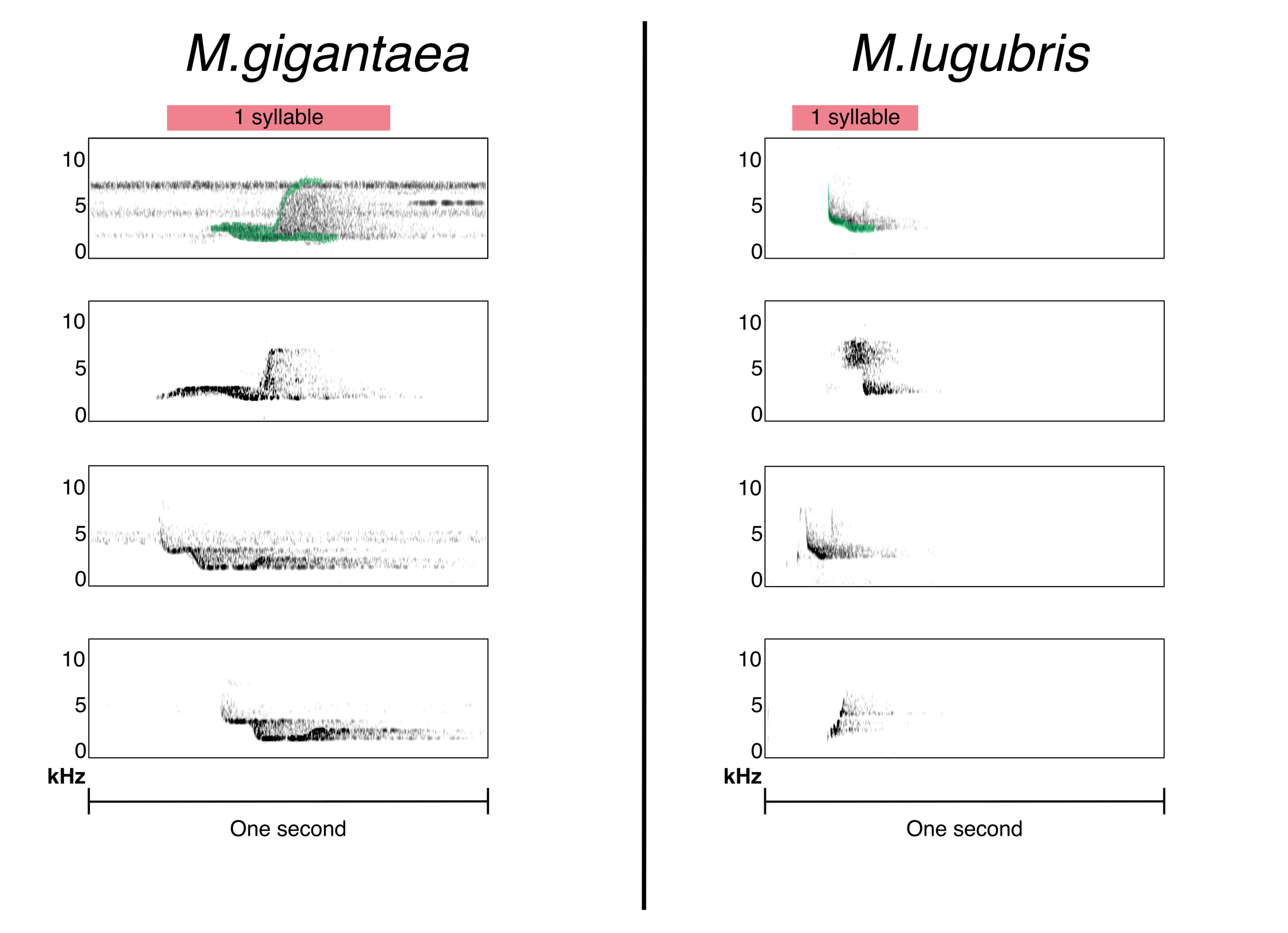

Supplement: S14 Fig — For each species, vocalisations from four different individuals are depicted. The top panel illustrates how vocalisations were measured in the acoustic software Luscinia: The user manually traces out the elements, the smallest unit within each vocalisation (in green), after which they are grouped into syllables (in red). Each vocalisation typically contains only 1 syllable for both species. The dynamic time warping algorithm in Luscinia creates a matrix of syllable dissimilarities using multiple frequency and time measurements that are extracted from these measured syllables. (TIF) [file pone.0293715.s014.tif]
