# Supplementary material for: Species-specific dynamics may cause deviations from general biogeographical predictions – evidence from a population genomics study of a New Guinean endemic passerine bird family (Melampittidae)
Source: PLoS One. 2024 May 23;19(5):e0293715. doi: 10.1371/journal.pone.0293715 (PMC11115331; doi:10.1371/journal.pone.0293715)
Supplement: S2 File — (DOCX) [file pone.0293715.s022.docx]

# Codes and parameters

**nf-polish**

**Removal of PCR duplicates:**

hts_SuperDeduper -1 $r1 -2 $r2 -f PE

Parameter explanation:

- -f PE: Output as paired-end fastq files

**Adapter trimming:**

trimmomatic PE -threads ${task.cpus} $r1 $r2 \ ${sample_id}_adapt_R1.fastq.gz ${sample_id}_1_u.fastq.gz \ ${sample_id}_adapt_R2.fastq.gz ${sample_id}_2_u.fastq.gz \ ILLUMINACLIP:${params.adapters}:2:30:10:8:TRUE

Parameter explanation:

- PE: Paired-end reads as input
- ILLUMINACLIP: Trim common Illumina adapters
- $params.adapters: A file containing a list of additional adapters that were used for sequencing (See S3 file)
- :2: Maximum number of mismatches
- :30: Palindromic match alignment score threshold
- :10: simple match alignment score threshold
- :8: minimum adapter length
- :TRUE: keep reverse complement read

**Read merging:**

pear -f $r1 -r $r2 -o ${sample_id} -p 0.0001 -v 20 -n 30 -k -j \ ${task.cpus}

Parameter explanation:

- -p 0.0001: maximum p-value to merge reads
- -v 20: minimum overlap size
- -n 30: minimum length of merged reads
- -k: keep unmerged reverse complement reads

**Quality trimming:**

trimmomatic PE/SE -threads ${task.cpus} $r1 $r2 \ ${sample_id}_qual_R1.fastq.gz ${sample_id}_1_u.fastq.gz \ ${sample_id}_qual_R2.fastq.gz ${sample_id}_2_u.fastq.gz LEADING:5 \ TRAILING:5 SLIDINGWINDOW:4:15 MINLEN:30

Parameter explanation:

- PE/SE: Paired-end / Single-end mode (only one should be specified)
- LEADING:5: Remove bases with quality below 5 from the beginning
- TRAILING:5: Remove bases with quality below 5 from the end
- SLIDINGWINDOW:4:15: Perform sliding window trimming (cut reads if the average quality within a window of length 4 falls below a threshold of 15)
- MINLEN:30: Minimum read length after trimming

**Low complexity read removal:**

remove_low_complex.py -1 $r1 -2 $r2 -u $u -c 0.5-p ${sample_id}

Parameter explanation:

- -c 0.5: If a base (A,T,C,G,N) occurs more than 50% within a read, it gets discarded

**nf-μmap**

**Mapping:**

bwa-mem2 mem $RefSeq $sample_file -t ${task.cpus} -R \ "@RG\\tID:${sample_id}\\tLB:${lib}\\tPL:ILLUMINA \

\\tPU:${params.rgPU}\\tSM:${SM}" > ${sample_id}.sam

Parameter explanation:

- -R: Add read group information (see <https://gatk.broadinstitute.org/hc/en-us/articles/360035890671-Read-groups> for a definition of each tag)

**Qualimap:**

qualimap bamqc -bam ${bam_file} -outdir ${sample_id}/ -outfile \ ${sample_id}_report.pdf -c \

--java-mem-size=${task.memory.toGiga()}G -nt ${task.cpus} -nw 500

Parameter explanation:

- -c: Paint chromosome limits within charts
- --java-mem-size: Set maximum Java heap size
- -nw 500: Number of windows (default 400, higher values decrease RAM usage)

**Mitochondrial phylogeny**

**Mitochondrial alignment:**

The alignment was generated using mafft *v7.407* [1] with the following parameters:

mafft --thread 16 --reorder --adjustdirection –globalpair \

--maxiterate 1000 $IN

Parameter explanation:

- -thread 16: Number of threads used
- -reorder: Order the output based on alignment similarity
- -adjustdirection: Generate reverse complements and align them
- –globalpair: Apply Needleman-Wunsch algorithm to generate a global alignment
- -maxiterate 1000: Maximum number of iterations. Recommended when

using -globalpair

**nf-phylo**

**IQ-TREE phylogeny on each window alignment:**

iqtree -s ${msa_fn} -m MFP -nt AUTO -ntmax ${task.cpus} –redo \

--prefix ${basename}

Parameter explanation:

- -s: Specify input alignment
- -m MFP: Perform extended model selection and subsequently use the best model for tree reconstruction
- -nt AUTO: Automatically detect the best number of threads based on the data and the computational environment
- -ntmax: Maximum number of threads
- -redo: Overwrite existing output files

**IQ-TREE phylogeny on all autosomes (concatenated windows) and each chromosome:**

iqtree -s ${msa_fn} -m GTR+I+G -B 1000 -nt AUTO \

-ntmax ${task.cpus} –redo --prefix ${prefix}

Parameter explanation (missing parameters are explained above):

- -m GTR+I+G: Infer tree underlying a GTR+I+G substitution model
- -B 1000: Number of bootstrap replicates

**ASTRAL3 phylogeny:**

java -jar astral.5.7.8.jar -i ${window_trees_fn} \

-o ${prefix}_ASTRAL.tree

Parameter explanation:

- -i: Specify input gene trees
- -o: Specify output file

**Site and window concordance factor (sCF + wCF) annotation using IQ-TREE:**

iqtree -t ${tree_fn} -s ${aln_fn} --gcf ${window_trees_fn} \

--scf 100 -T ${task.cpus} --prefix ${prefix}

Parameter explanation:

- -t: specify a file containing a starting (species) tree
- --gcf: compute gene (here window) concordance factor, provide a file with all window trees
- --scf 100: sample 100 random quartets around each internal branch to compute site concordance factors based on parsimony

**ANGSD**

Beagle files were generated using ANGSD *v0.938* [2] using these parameters:

angsd -bam Bams.list -out $OutPath -doSaf 1 -GL 1 -doGlf 2 \

-doMajorMinor 1 -ref $REF -anc $REF -doMaf 1 -minMaf 0.05 \

-SNP_pval 1e-6 -doCounts 1 -setMinDepth 62 -setMaxDepth 465 \

-minInd 15 -minQ 20 -minMapQ 20 -uniqueOnly 1 -only_proper_pairs 1 \ -remove_bads 1 -baq 1 -C 50 -P 10

Parameter explanation:

- -bam bamlist.txt: Specify we're using bam, give the name of the bamlist
- -out: set prefix for output files
- -doSaf 1: Calculate the Site allele frequency likelihood based on individual genotype likelihoods assuming Hardy-Weinberg-Equilibrium
- -GL 1: Estimate genotype likelihoods using SAMtools model
- -doGlf 2: Generate beagle input file
- -doMajorMinor 1: Infer major and minor from genotype likelihoods
- -ref $REF: Give reference sequence
- -anc $REF: Give ancestral sequence, as we did not have one available, we used the reference sequence, but following steps need to be performed with -fold 1
- -doMaf 1: Estimate allele frequencies with known major minor
- -minMaf 0.05: Only work with sites with a minor allelic frequency above 0.05
- -SNP_pval 1e-6: Test for polymorphic sites and only output the ones which have a likelhood ratio test p-value < 1e-6
- -doCounts: Output the counts of the different bases
- -setMinDepth 62: Discard sites if their total sequencing depth (all individuals added together) is below the given value. As an example I chose 62 (2*31) to get on average at least 2 reads per site per individual for this dataset (n = 31 individuals)
- -setMaxDepth 465: Discard sites if their total sequencing depth (all individuals added together) is above the given value. E.g. for the same subset 465 (31*15), the factor is an arbitrary decision, which should removes repetitive sites that can cause excessive depth
- -minInd 15: Only keep sites with at least minIndDepth (default is 1) from at least the given number of individuals, I chose 15 to include at least half of the individuals
- -minQ 20: Minimum allowed base quality score
- -minMapQ 20: Minimum allowed mapping quality score
- -uniqueOnly 1: Remove reads that have multiple best hits.
- -only_proper_pairs 1: Include only proper pairs (pairs of read with both mates mapped correctly)
- -remove_bads 1: Same as samtools’ -x flag which removes reads with a flag above 255 (not primary, failure and duplicate reads)
- -baq 1: Perform base alignment quality (BAQ) computation. Reduces false SNP calling at possibly misaligned bases [3]
- -C 50: Adjust mapping quality among reads with high numbers of mismatches. For reads mapped with BWA, a value of 50 is recommended (according to samtools’ documentation)
- -P 10: Number of threads to be used

**PCA**

Covariance matrices for PCAs were generated using PCANGSD [4]

pcangsd.py -beagle ${InPATH}/${i}.beagle.gz -threads 8 \

-o ${OutPATH}/${i}

Parameter explanation:

- -beagle: Specify input beagle file
- -threads 8: Number of cores to be used
- -o: Specify output directory and name

**NGSAdmix**

Admixture proportions were estimated through NGSAdmix [5] with the following script:

#Loop through values of k

for k in $(seq 1 $MaxK)

do

#Loop through number of replicates (also used as seed)

for n in $(seq 1 $MaxReps)

do

angsd/misc/NGSadmix -likes ${InPATH}/${i}.beagle.gz -seed ${n} \

-K ${k} -P 4 -o ${OutPATH}/${i}/${i}_k${k}_r${n}

done

done

Parameter explanation:

- -likes: Input file in .beagle format
- -seed ${n}: Initial seed to be used in the expectation-maximisation (EM) algorithm. The number of replicate (e.g 1-10) is being used as seed.
- -K ${k}: Number of ancestral populations
- -P 4: Number of threads
- -o: Output path and prefix

**Heterozygosity estimates**

To obtain estimates of individual heterozygosity, we first generated sample allele frequency (saf) files using ANGSD with the following settings:

angsd -i $bam -out $OutPath/${filename} -doSaf 1 -GL 1 \

-doMajorMinor 1 -ref $REF -anc $REF -doMaf 1 -doCounts 1 \

-setMinDepth 2 -setMaxDepth 15 -minQ 20 -minMapQ 20 -uniqueOnly 1 \ -only_proper_pairs 1 -remove_bads 1 -baq 1 -C 50 -P 10

Parameter explanation (missing parameters are explained above under ANGSD):

- -i: Specify single input .bam file

In the next step, .saf files were converted into global site frequency spectra (sfs):

angsd/misc/realSFS ${i} -P 16 -fold 1 > ${OutPATH}/${filename}.sfs

Parameter explanation:

- -fold 1: Generate a folded site frequency spectrum. Necessary when no ancestral sequence is available and the reference is used in its stead
- -P 16: Number of threads

**Thetas estimation:**

SAFs were generated the same way as for NGSAdmix, but filters for p-value or MAF were left out, the applied parameters were as follows (see above for explanations):

angsd -bam Bams.list -out $OutPath -doSaf 1 -GL 1 -doGlf 2 \

-doMajorMinor 1 -ref $REF -anc $REF -doMaf 1 -doCounts 1 \

-setMinDepth 62 -setMaxDepth 465 -minInd 15 -minQ 20 -minMapQ 20 \

-uniqueOnly 1 -only_proper_pairs 1 -remove_bads 1 -baq 1 -C 50 -P 10

SFS were generated with the same command as for single individuals:

angsd/misc/realSFS ${InPATH}/${i}.saf.idx -fold 1 \

-P 12 > ${OutPATH}/${i}.sfs

Chromosome-wide theta estimates were obtained using the following commands:

angsd/misc/realSFS saf2theta ${i}.saf.idx -sfs ${i}.sfs -fold 1 \

-P 1 -outname ${OutPATH}/${i}

angsd/misc/thetaStat do_stat ${OutPATH}/${i}.thetas.idx

Parameter explanation:

- -fold 1: Generate a folded site frequency spectrum. Necessary when no ancestral sequence is available and the reference is used in its stead
- -P 1: Number of threads
- -outname: Specify output path and prefix

Additionally, we obtained window-based thetas with the following command:

angsd/misc/thetaStat do_stat ${OutPATH}/${i}.thetas.idx -win 10000 \ -step 20000 -outnames ${OutPATH}/${i}.Win

Parameter explanation:

- -win 10000: Define sliding window size
- -step 20000: Define step size in which windows are moved
- -outnames: Specify output path and filename

**D_xy_ calculations:**

The first step was to create a maf file that contains all individuals/populations of interest. This will provide a list of sites to analyse even if a certain population has a fixed allele at a site. The following settings were used:

angsd -bam Bams.list -out $OutPath -GL 1 -doMajorMinor 4 -ref $REF \ -anc $REF -doMaf 1 -SNP_pval 1e-6 -doCounts 1 -setMinDepth 58 \

-setMaxDepth 435 -minInd 14 -minQ 20 -minMapQ 20 -skipTriallelic 1 \ -uniqueOnly 1 -only_proper_pairs 1 -remove_bads 1 -baq 1 -C 50 -P 10

Parameter explanation:

- doMajorMinor 4: Force major to be the same as in the provided reference, minor inferred from GL
- -skipTriallelic 1: Don’t include triallelic sites

Using the obtained sites from the previous step, per-population maf files could now be generated:

angsd -bam Bams.list -out $OutPath -sites $REG_FILE -GL 1 \

-doMajorMinor 4 -ref $REF -anc $REF -doMaf 1 -doCounts 1 \

-minInd 3 -minQ 20 -minMapQ 20 -skipTriallelic 1 -uniqueOnly 1 \

-only_proper_pairs 1 -remove_bads 1 -baq 1 -C 50 -P 8

Parameter explanation:

- -sites: limit analysis to only specific sites, the provided file was tab separated and contained information on the chromosome/scaffold name, the position of the site as well as the major and minor allele

# References

1. Katoh K, Standley DM. MAFFT multiple sequence alignment software version 7: improvements in performance and usability. Mol Biol Evol. 2013;30(4):772–80.

2. Korneliussen TS, Albrechtsen A, Nielsen R. ANGSD: Analysis of Next Generation Sequencing Data. BMC Bioinformatics. 2014 Nov 25;15(1):356.

3. Li H. Improving SNP discovery by base alignment quality. Bioinformatics. 2011 Apr 15;27(8):1157–8.

4. Meisner J, Albrechtsen A. Inferring Population Structure and Admixture Proportions in Low-Depth NGS Data. Genetics. 2018 Oct 1;210(2):719–31.

5. Skotte L, Korneliussen TS, Albrechtsen A. Estimating Individual Admixture Proportions from Next Generation Sequencing Data. Genetics. 2013 Nov 1;195(3):693–702.
