# Supplementary material for: Species-specific dynamics may cause deviations from general biogeographical predictions – evidence from a population genomics study of a New Guinean endemic passerine bird family (Melampittidae)
Source: PLoS One. 2024 May 23;19(5):e0293715. doi: 10.1371/journal.pone.0293715 (PMC11115331; doi:10.1371/journal.pone.0293715)
Supplement: S3 File — (DOCX) [file pone.0293715.s023.docx]

>PrefixPE/1

TACACTCTTTCCCTACACGACGCTCTTCCGATCT

>PrefixPE/2

GTGACTGGAGTTCAGACGTGTGCTCTTCCGATCT

>PE1

TACACTCTTTCCCTACACGACGCTCTTCCGATCT

>PE1_rc

AGATCGGAAGAGCGTCGTGTAGGGAAAGAGTGTA

>PE2

GTGACTGGAGTTCAGACGTGTGCTCTTCCGATCT

>PE2_rc

AGATCGGAAGAGCACACGTCTGAACTCCAGTCAC
