# Supplementary material for: Species-specific dynamics may cause deviations from general biogeographical predictions – evidence from a population genomics study of a New Guinean endemic passerine bird family (Melampittidae)
Source: PLoS One. 2024 May 23;19(5):e0293715. doi: 10.1371/journal.pone.0293715 (PMC11115331; doi:10.1371/journal.pone.0293715)
Supplement: Attachment — Submitted filename: ResponseToReviewers.docx [file pone.0293715.s024.docx]

**Reviewer #1:** *Interesting study, in which the authors seem to have managed to produce good quality data from museum samples. However, in terms of the analyses, and interpretation and presentation of results, the study/paper needs in my opinion substantial improvement. See my comments below. The major comments are indicated with 'MAJOR'.*


**Abstract:**
*MAJOR: 50% of the abstract is intro, 30% methods, with effectively only one sentence describing the results. If in the conclusion you claim that certain populations should be raised to species level, this should be mentioned in the abstract. Also, refer back to the title: which species-specific dynamics? And how do the study species deviate?*

RESPONSE: We have revised the abstract to accommodate this critique. Specifically, we have shortened the intro and added information about the potential elevation of Lesser Melampitta populations to full species. We also refer back to the title.

**Introduction**
Line 68-70: *what would young lineages be better adapted to lowland habitats?*

RESPONSE: This sentence summarises the concept of taxon cycles in which taxa go through phases of expansions (lowland generalists) and contractions (highland specialisation). The idea is that from time-to-time lowland generalists colonise new islands. As they are generalists, they tend to colonise peripheral (coastal) habitat. Over time these taxa move inland and upwards and become specialists. Little is known about the order of this. In any case, when it comes to taxon cycles, the new colonisers are thought to be “pioneer species” that can exploit the unstable coastal habitats better than more specialised old species. We have tried to summarise this very briefly in the introduction and have now added the word “generalist” to the text to indicate that the new lowland colonisers are expected to be generalists.

Line 85: *why ‘in accordance’? Why do you assume that the Lesser M. is an old lineage?*

RESPONSE: The family Melampittidae with only the two species investigated herein represent a very deep linage within Corvides and is one of a few very distinct and old lineages (16.1 My) in New Guinea. Moreover, the two currently recognised species within this family are placed in different genera and diverged from each other about 10 Mya. Although old is a relative term, these two genera represent old linages within Corvides and the divergence between these two species/genera is also old compared to other sister taxa divergences in this region.

Line 91-93: *Are mid-elevations to be considered here as ‘lowland’?*

RESPONSE: We apologise that this was not clear from the text, but we do not consider the habitat of *M. gigantaea* as lowlands, but neither as high elevations. We wanted to point out that this species has moved out of the coastal lowlands to some extent, but not into the high elevations that we normally observe for old/relictual taxa (as frequently observed in other avian groups on New Guinea). We consider here a scenario where rather than moving even higher up, *M. gigantaea* moved into suboptimal karst habitats (at mid-elevations) where they would also encounter less competition.

Line 95-98: *specify here number of individuals sequenced, and to which depth. Also specify roughly which kind of analyses.*

RESPONSE: This is a valid point and we have added this information to the introduction. More detailed numbers can be found at the beginning of the results and in supplementary table S1.

Line 96: *MAJOR: while genomic data allows indeed to determine present-day population structure with the methods employed in this study (pca, phylogeny, admixture), but more methods are needed to determine how ‘habitat connectivity across space and time has shaped differentiation’*

RESPONSE: Thank you for this important note. We have deleted and rephrased this segment to be more precise in the aim of this study and which questions can be answered with the applied methodology.

Line 99-106: *Wouldn’t you expect a priori the opposite? Namely, the high-elevation species (Lesser. M) to have low connectivity between mountain-tops and hence to have population structure?*

RESPONSE: Intuitively, we agree that we would expect a high-elevation species to be more isolated across a mountain range than a species inhabiting lower elevations. However, in this study - due to the large distances between the few distributional data points - *M. gigantaea* was expected to exhibit a stronger structure between populations. Additionally, since *M. gigantaea* is specialised on karst environments, we would expect the species to struggle outside these environments and be strongly isolated.

**Methods**
Line 109-117: *why so few modern samples? Are samples difficult to acquire?*

RESPONSE: Yes, the low number of modern samples is due to the difficulty of collection. For example, co-author Knud Jønsson spent 6 months in the field in Papua New Guinea within the distribution of *M. lugubris* and only ever collected two *M. lugubris* samples. Additionally, sampling in Indonesia has become almost impossible due to government regulations. For *M. gigantaea* sampling is even more difficult as we were able to obtain the only fresh sample collected within the last 50 years as well as all 6 historical specimens that are available in museum collections worldwide.

Line 126: *2x100bp read length is deliberate?*

RESPONSE: We choose a read length of 2x100 bp in our museomics projects as our fragments rarely reach sizes longer than 200 bp (the average length of our fragments ranges from about 90 bp to 130 bp, see e.g. Irestedt et al. (2022)) that would make it worthwhile to sequence with the 2x150 bp chemistry.

Irestedt, M., Thörn, F., Müller, I. A., Jønsson, K. A., Ericson, P. G., & Blom, M. P. (2022). A guide to avian museomics: Insights gained from resequencing hundreds of avian study skins. Molecular Ecology Resources, 22(7), 2672-2684.

Line 127-128: *How much data per sample? Expected genome size, targeted depth?*

RESPONSE: We have added the requested information to the text. Our expected genome size was about 1 GB, given that the genome sizes in passerine birds is conserved and by the size of our chosen reference genome (*Corvus cornix*, genome size: 1.031 GB). As we did not sequence any *de novo* genome of Melampittidae we do not have a more precise estimate.

Line 129-138: *While I appreciate the efforts made to make the work reproducible using a pipeline, the authors still need to describe here the settings of each software used by the pipeline (e.g., samtools and bwa). For instance, which base quality, mapping quality, and alignment score thresholds were used? Furthermore, all software used by the pipeline, needs to be mentioned here, and referenced.*

RESPONSE: Thank you for the comment. We have added each tool and its version of all tools that are part of the Nextflow workflows. All implemented flags are now included in supplementary file S2. Bwa-mem2 and samtools were both run with default settings as we perform the relevant filtering steps in subsequent downstream analyses anyway.

Line 142: *Because the authors so far did not mention the mean sequencing depth, it is hard to evaluate whether these thresholds are appropriate. The mitogenome depth is usually much higher than the nuclear depth, and hence 20x could be low. Could the authors present a summary of mean coverage of both the nuclear and mito genome?*

RESPONSE: We have expanded supplementary table S1 to distinguish depth-of-coverage (DoC) of the nuclear genome and the mitochondria. The median DoC 786 *x* of the mitochondria is of course much higher than in the nuclear genome, a minimum DoC of 20 *x* is in our opinion still sufficient to make confident variant calls with low biases from erroneous reads.

Line 152: *Mitochondrial genomes (!) might be effectively haploid, but in reality they are highly polyploid: each cell contains hundreds or thousands of copies which are independently proliferating, which may also cause heterozygous calls.*

RESPONSE: This is absolutely true and a valid point. While we expect a certain number of heterozygous calls due to heteroplasmy, we saw a significantly higher number of such sites in six individuals (> 50 sites although most of these had more than 100-200 heterozygous sites, compared to 0-5 sites in most other individuals) and these sites were always concentrated in the same region across these individuals. As the flanking regions of the reads that produce these blocks were not of mitochondrial origin, we consider it highly likely that they represent NUMTs and has thus been deleted. We would not expect such a concentrated pattern due to random mutations in independent mitochondria especially not if only occurred in some individuals.

Line 155: *for museum samples, a depth below 4x is not too bad. Why freebayes? Other genotyping software (like bcftools, GATK or ANGSD) have been reported to have higher accuracy.*

RESPONSE: A depth of coverage around 4 *x* is indeed not too bad, but it’s also not great in comparison to coverages typically seen for sequencing data of fresh tissue samples. Genotyping software such as GATK was developed for analysing data from model organisms where we often have a good reference point to calibrate our variant calling. In other words, we can optimise our parameter settings for variant call recalibration using truth and training sets. However, this is much more difficult to do for non-model organisms and for historical datasets where there is a higher error rate. At the same time, 4 *x* depth of coverage is indeed much higher than typically seen for ancient DNA data where genotype likelihood methods such as employed by ANGSD excel. We therefore opted for a holistic approach using both ANGSD (for admixture analyses, PCAs and estimates of π, Tajima’s D, Heterozygosity) as well as freebayes, because the latter allows the use of a population prior when variant calling. This population information is used for the imputation of genotypes across a population and should therefore limit potential residual deamination patterns in historical samples. We are unaware of any reports that have demonstrated a higher accuracy for other genotyping software with this kind of datasets.

Line 160-163: *Again, it does not suffice to only mention the pipeline. Even though this information could be looked up at the Github page, it needs to be specified here which settings were used to run IQtree2 and ASTRAL.*

RESPONSE: Thank you, we have now expanded this section to mention all major steps and tools citing the respective creators. We have also modified supplementary file S2 to include all implemented flags and filters.

Line 162: *Running IQtree (maximum likelihood approach) on an input dataset of SNPs (diploid, recombining loci), violates underlying assumptions of ML tree building.*

RESPONSE: We don’t think that a concatenated dataset violates the underlying assumptions of ML tree building itself. In fact, this is a very common approach to infer the prevailing phylogenetic signal across a (whole-genome) dataset (see e.g. Lopes, F., et al. *Systematic Biology* (2021), Bein, B., et al. *Marine Biology* (2023)). However, we do agree that it does not account for any possible variation in coalescent histories between loci across and between chromosomes (i.e. where there has been a recombination event). We therefore complement the ML inference of concatenated data with a summary-coalescent species tree estimation based approach (ASTRAL3) which is based on genomic windows of different lengths (here we ran 2000, 5000, 10000 and 20000 bp windows) rather than concatenation.

Lopes, F., Oliveira, L. R., Kessler, A., Beux, Y., Crespo, E., Cárdenas-Alayza, S., ... & Bonatto, S. L. (2021). Phylogenomic discordance in the eared seals is best explained by incomplete lineage sorting following explosive radiation in the southern hemisphere. *Systematic biology*, *70*(4), 786-802.

Bein, B., Lima, F. D., Lazzarotto, H., Rocha, L. A., Leite, T. S., Lima, S. M., & Pereira, R. J. (2023). Population genomics of an Octopus species identify oceanographic barriers and inbreeding patterns. *Marine Biology*, *170*(12), 161.

Line 162: *Better to use here the word ‘locus tree’ rather than ‘gene tree’*

RESPONSE: You are correct, since we are doing a window-based the term gene tree is not correct here. In the rephrased segment we have avoided this term.

Line 165: *MAJOR: which of the below mentioned statistics estimate levels of differentiation? In reality, all statistics (nucleotide diversity, heterozygosity and SFS) estimate genetic diversity (with Tajima’s D testing for neutrality). PCA and admixture show population structure, but do not estimate the level of population differentiation.*

RESPONSE: Thank you very much for pointing out this issue. We realise that the term differentiation was not used correctly here as we haven’t employed a direct measure of it. We have corrected all mentions of differentiation to either refer to population structure or genetic diversity.

Line 170: *MAJOR: With only 6 individuals per population, does it really make sense to try to reconstruct the SFS?*

RESPONSE: While the sample size is close to the recommended minimum for population level estimates, we still believe that our sampling scheme allows for a decent estimate of our calculated statistics as we included all available samples of *M. gigantaea* which cover its entire distribution across New Guinea as well as *M. lugubris* individuals that all cover different localities within the distribution of its populations.

Line 173: *Admixture analyses were run…*

RESPONSE: Thank you for pointing out this grammatical error, we have now corrected it in the text.

Line 178: *MAJOR: Does it really make sense to calculate Tajima’s D for an entire chromosome? This test is designed for a single, non-recombining locus.*

RESPONSE: This is absolutely correct, the original calculations for Tajima’s D were of course based on per-site estimates that we then averaged over each chromosome. Our main reason for looking at Tajima’s D was to see whether we also see a signal that could arise due to population expansion or contraction, so we only presented it as a genome-wide comparison between the two species. For completeness, we have now updated supplementary figure S3 to show a window-based visualisation of Tajima’s D across each chromosome.

**Results**
Line 232-247: *move to methods section.*

RESPONSE: We have now moved this segment to the end of the methods section “DNA sampling, sequencing and read processing”

Line 295-317: *a population split of 4-5 Mya, would this not imply these populations are in fact different species? Else, is it possible that the PSMC settings inflate the divergence time estimate?*

RESPONSE: We do not think the divergence times obtained through hPSMC to be an overestimate as we also complemented the estimates based on mitochondrial divergence. We agree that such deep splits are indicative of species level divergence, which we also suggest at the end of the discussion. Although, to explicitly state that these populations are independent species, our opinion is that other types of evidence, such as morphological and vocal variation, should also be examined. However, this is beyond the scope of this paper.


**Discussion**
Line 340: *how do we know it is an old lineage? When does a lineage quality as ‘old’?*

RESPONSE: See above (Line 85).

Line 349: *MAJOR: a population split of 4-5 Mya, would this not imply these populations are in fact different species? Else, is it possible that the PSMC settings inflate the divergence time estimate?*

RESPONSE: See previous response (Line 295-317)

Line 368: *See my previous comment for line 99-106: Wouldn’t you expect a priori the opposite? Namely, the high-elevation species (Lesser. M) to have low connectivity between mountain-tops and hence to have population structure?*

RESPONSE: See response to line 99-106. On a relative scale, we would expect *M. gigantaea* to be even more isolated due to its habitat specificity and the large distance between each habitat.

**Conclusion:**
Line 396: *Which intrinsic properties?*

RESPONSE: We have modified this sentence to clarify that we refer to the recent population decline in *M. gigantaea*

Line 399-401: *MAJOR: Perhaps I overread, but how did you estimate levels of divergence, other than using hPSMC to estimate divergence times? As mentioned in a previous comment, a split time of 4-5 Mya would indeed suggest species status. But to be better able to evaluate this claim, it would be useful if the authors would estimate levels of genetic divergence between the populations (i.e. Dxy).*

RESPONSE: See our response to line 295-317. In addition to hPSMC we also obtained rough estimates from mitochondrial divergences and the average rate of divergence in avian mitochondria. Ideally, we would of course want to implement better methods such as d_xy_ but due to the low number of individuals within *M. gigantaea* we could not compare levels of divergence between different populations within this species.

**Figures**

Figure 1. *MAJOR colour coding of eastern populations does not correspond between A and B. Add to the figure legend (following the species names) the elevation ranges of the two species. Throughout the text, the authors could make clearer (or reminder the reader) that the Lesser M. occurs at high elevation, and the Greater M at mid elevation. In the caption of figure 1, specify which software/method has been used to construct the phylogeny.*

RESPONSE: We have updated this figure to subdivide the Eastern populations as we did in the phylogeny. We have also added the elevational distribution of each species to the legend and updated the figure caption.

Figure 2. *MAJOR These analyses only qualitatively assess population structure, but do not provide quantitative estimates (e.g. Dxy or Fst). The legend (e.g. Vogelkop, Weyland, etc) does not correspond with the labels of the admixture plot. Regarding the admixture plot: I find it suspicious that there is no evidence for admixture whatsoever. Especially between the central populations, which are geographically close to each other, should there not be any exchange? Regarding the He-plot, could you present He per population (e.g., west, east, central) rather than per species?*

RESPONSE: See our response for line 165, we have corrected the caption to avoid the term differentiation. We have fixed plot A to use the same labels as Figure 1 and Figure 2B. Due to the long divergence time (4-5 mya between Western+Central and Eastern) between *M. lugubris* populations, we believe that the lack of admixture would further support our proposition to consider these as in fact different species. This long-term isolation could e.g. be driven by the deep valley separating the central and eastern populations (see Fig. 1). We have added a supplementary figure (S13) to show the heterozygosity between not only the species, but all populations of *M. lugubris*.

Figure 3. *MAJOR Since PSMC is used to evaluate split times, why not plot the curves on top of each so that it is better possible to evaluate when the lines diverge? It should also be made more clear which populations/species each tile represents (the sample names are not informative). Where are the bootstraps? In figure 3c, one of the samples need to be corrected, in order to overlap (which they clearly do, just need to be corrected for difference in depth). Where is the hPSMC plot?*

RESPONSE: This figure specifically was to show only the population history of each species/population and it includes more individuals than the hPSMC. hPSMC was only run on the single highest individual from each species/population. We have updated the figure legends, added bootstraps to these plots (we had to split the last panel as the bootstraps would make the plot too messy when overlapping 6 individuals) and adjusted the FNR calculation to remain consistent between pairs (see lines 226-230). The hPSMC plots are part of the supplementary material (S6-S10).

**Reviewer #2:** *This study provides a novel genetic analysis of a so-far poorly-studied passerine bird family (Melampittidae). It is a huge merit of the team that they managed to analyse all seven known museum specimens of Megalampitta gigantaea. Although this species is currently not considered to be under threat (least concern, IUCN), there is a considerable knowledge gap on that species’ distribution and intraspecific diversification (e.g. whether it comprises more than one evolutionary significantly unit). This emphasizes the relevance of museomics for current biodiversity research. The research team is known for their strong expertise of collection-based genetics/genomics and the use (and development) of specific protocols for historic DNA analysis. The results shade new light on the cryptic diversification in this group, e.g. despite being considered a monotypic species M. lugubris turned out to comprise several distinct genetic lineages restricted to different mountain ranges on New Guinea. This is a fine study that is highly recommendable for publication. In the following I am commenting on a few aspects of the study that should be outlined or explained in a little more detail in a revised manuscript.*


l. 63: *“older taxa are often found at higher elevations, while young lineages that are generally widespread, good dispersers and show little differentiation inhabit the lowlands”. Following a general introduction on mountain biodiversity and evolutionary patters in mountain specialists, this reads as if it was a generalized statement. However, this seems to be a rather characteristic pattern on New Guinea (see l. 71), “island systems” (l. 65) or in other tropical ecosystems, whereas in other big mountain systems of the Earth, this does not seem to be the common pattern. For the Himalayas (and the Andes) see Fjieldsa et al. (2012: Fig. 1) with both mountain systems showing highest richness of both youngest and oldest species. Furthermore, for the Himalayas Price et al. (2014) stated that „we found that the average age of separation of species in the assemblage declines monotonically with elevation, rather than being lowest in the most species-rich elevational band”. I think in this introductory paragraph it is important to emphasize that it is not the general pattern for any mountain system that evolutionary ancient species occur on top of the mountain.*

RESPONSE: Thank you for this important comment, we have clarified that this pattern has mainly been observed on mountainous islands.

l. 82: *“recent genetic results have placed the family as sister to crows (Corvidae) and shrikes (Laniidae) with an estimated divergence time from these at ca. 17 Mya [31]”
This statement refers to Oliveros et al. (2019), however, this year a new study has been published by
McCollough et al. 2023: Ornithology, 2023, 140, 1–11 https://doi.org/10.1093/ornithology/ukad025. Their phylogeny based on UCEs placed Eurocephalidae as the sister to Corvidae, Platylophidae sister to Laniidae and Melampittidae was sister to all four. For completeness, this study should be acknowledged here.*

RESPONSE: Thank you very much for pointing out this updated phylogeny. We have included the reference and modified the text in this sentence.

l. 115: *“the work is mainly based on old museum specimens for which the Nagoya Protocol does not apply.” I do not expect a reply from the authors to this comment, because this is just a friendly, but to my feeling important reminder. I would like to send out a warning to the authors rather not to use such vague statements. The word “mainly” implies that “not all” samples used are old and could evoke the impression of decision makers (including the persons in charge on the NFPs of a country of origin), that for some of the material the Nagoya Protocol might theoretically apply and that this must have been checked before publication (even before performing any genetic work on the material). Moreover, I think this statement is unnecessary, because at least to my information Papua New Guinea (PNG) is not a signatory country of the Nagoya Protocol (check here)
https://www.cbd.int/abs/nagoya-protocol/signatories/ whereas, nevertheless PNG has established an NFP for the Nagoya Protocol. So, this is one of the many very tricky cases and potential pitfalls for us scientists (check here) https://www.cbd.int/countries/?country=pg*

*I assume that even the two “fresh tissue samples” (l. 111) had been assessed before October 2014; if so, then I would suggest either stating that Nagoya does not apply to the study material, because all samples were assessed before that date – however, in that case, that remark is rather unnecessary and I would recommend rather deleting this statement, if journal policies do not require adding a disclaimer on compliance with the Nagoya Protocol.*

RESPONSE: Thanks for the heads up. We are well aware of the Nagoya protocol. As you mention PNG has not signed it. Nevertheless, we always make sure to have permits (now explicitly mentioned in the acknowledgements) and to include local people in all aspects of the work.

l. 117: *“permits are available”; it would be good to cite these (including permit numbers) in the acknowledgements (collecting or research permits in the country of origin etc.).*

RESPONSE: Yes, and we are including these now.

l. 207: *“We applied the simple 2% rule…” Indeed, this is a very simplistic approach, and I am not sure whether I correctly understand, how this was actually done. I think we can infer from the reference to Weir & Schluter (2008) that the empirical cytochrome-b rate was apparently applied across the entire mitogenome. To me it seems that pairwise distances (inferred from whole mitogenomes including tRNAs, rRNAs and non-coding regions like the D-loop) among taxa/clades simply were transferred into split ages using the cytb rate (the statement in l. 205/206 evokes this impression). Considering that mitochondrial genes evolve a different substitution rates (in fact quite different between coding and non-coding regions; ; compare Lerner et al. 2011: Curr Biol 21, 1838-1844), this is not a very precise approach (and I would not consider this the state of the art). The more convincing method would be reconstructing a time-calibrated mitochondrial phylogeny, using for example the thirteen coding-markers of the mitogenome (as thirteen separate partitions in BEAUti) and applying empirical rates to each partition (e.g. from Lerner et al. 2011). If the simple approach is preferred, then it should be limited to the cytb fragments of the Melampittidae mitogenomes, because the Weir & Schluter estimate refers only to cytb.*

RESPONSE: Thank you very much for this important comment. We are aware of the drawbacks of applying the rather general 2 % rule. We merely applied the 2% rule to the mitochondrial dataset to verify our estimates obtained through hPSMC. Nonetheless, we have corrected the rate by instead taking the average rate of 1.8 % obtained for the different mitochondrial regions and genes following Lerner et al. (2011) to at least use a slightly more precise estimate for the entire mitochondrial divergence. This has very minor influence on our divergence time estimates and the conclusions of the paper

l. 224: *“Elements were measured as continuous sound traces and then grouped into syllables within each vocalisation (each vocalisation contained only one syllable).”
It is unclear to me what was done here. From that scarce information I think it is not possible to infer, how song characters were quantified, i.e. which sound parameters (frequency, time) were actually measured. Maybe this information is hidden in the statement on the DTW algorithm and the link to reference 62 (l. 226/227). Nevertheless, this vague information is not helpful to understand the differences in songs among the two species (except that one is more variable than the other).*

RESPONSE: Thank you for pointing this out, we understand that this information is crucial for the reader to follow. We have accordingly expanded on the methods sections, which we hope will now make this section clearer. The DTW algorithm makes use of multiple acoustic parameters- different frequency measurements, the shape of the frequency curve, time, etc. to create an overall matrix of syllable dissimilarities (Lachlan 2016).

Lachlan, R.F. (2016). Luscinia: a bioacoustics analysis computer program. rflachlan.github.io/Luscinia.

*If 10 principal components were extracted these should normally correlate with song parameters (to be inferred from factor loads). So, as the reader I would like to know: What does differentiation along PC1 actually tell us? (Fig. 4; ~ 44% of variation) Which acoustic traits correlate with PC1? Are these differences in pitch, speed, complexity? Given that many acoustic features change with habitat density that might also relate to the two study species (mountain forests versus [more open?] karst habitats), this seems relevant information to me. This should be outlined in much more detail.*

RESPONSE: This is also a very important point made and we hope that the methods now make it clearer as to why we could not give more details into which acoustic parameters correlate with the different PC’s. The final output of the DTW analysis is a syllable dissimilarity matrix which is non-linear. Therefore, in order to conduct further analysis on it (and for plotting), we converted the matrix into Euclidean dimensions using non-metric multidimensional scaling (NMDS) (this is an in-built feature in the acoustic software Luscinia (Lachlan 2016), and is a standard procedure in many acoustic studies (Holveck et al., 2008; Verzijden et al., 2010; Edelaar et al., 2012; Boogert et al., 2018; Roper et al., 2018, among othersThe components that NMDS produces is arbitrary and therefore does not correlate directly to any acoustic features. The components therefore simply correlate to an average value of acoustic dissimilarity between syllables, having accounted for the multiple acoustic features that the DTW process took into consideration (Lachlan 2016).

Holveck, M. J., Vieira de Castro, A. C., Lachlan, R. F., ten Cate, C., & Riebel, K. (2008). Accuracy of song syntax learning and singing consistency signal early condition in zebra finches. *Behavioral Ecology*, *19*(6), 1267-1281.

Verzijden, M. N., Van Heusden, J., Bouton, N., Witte, F., ten Cate, C., & Slabbekoorn, H. (2010). Sounds of male Lake Victoria cichlids vary within and between species and affect female mate preferences. *Behavioral Ecology*, *21*(3), 548-555.

Edelaar, P., Alonso, D., Lagerveld, S., Senar, J. C., & Björklund, M. (2012). Population differentiation and restricted gene flow in Spanish crossbills: not isolation‐by‐distance but isolation‐by‐ecology. *Journal of Evolutionary Biology*, *25*(3), 417-430.

Boogert, N. J., Lachlan, R. F., Spencer, K. A., Templeton, C. N., & Farine, D. R. (2018). Stress hormones, social associations and song learning in zebra finches. *Philosophical Transactions of the Royal Society B: Biological Sciences*, *373*(1756), 20170290.

Roper, M. M., Harmer, A. M., & Brunton, D. H. (2018). Developmental changes in song production in free-living male and female New Zealand bellbirds. *Animal Behaviour*, *140*, 57-71.

*A figure showing sonagrams would be actually helpful, because from the text I have no idea of the specific vocalizations (traits measured could then also be shown in one of the sonagrams). A closer check of sonagrams, could also be helpful for interpretation of the “significant outlier” in M. lugubris (legend of fig. 4; page 14)C. What means an “odd vocalization” in this context? Given the apparently high uncertainty of the context of a recorded vocalization (as outlined in Acoustic recordings and analysis), this one vocalization might not even be homologous to the other vocalizations of M. lugubris. Considering that even the distinction between “calls” and “songs” was unclear in this study, the vocalizations in general should be described and illustrated in much more detail.*

RESPONSE: This is absolutely correct! We have added a figure depicting the sonograms as supplementary figure (S14). However, a closer look at sonograms does not aid us in interpreting the ‘odd vocalisation’ as mentioned in the legend to Fig. 4, due to uncertainty in sampling method and lower number of samples. Therefore, the sample may not even be of the same type as the other vocalisations, as the reviewer points out. We include it here though, because we feel uncomfortable about excluding it since we have the same level of contextual information about all the vocalisations and also may not have sampled the entire vocal repertoire of the species. We would like to stress to the reviewer, that the acoustic analysis is merely preliminary and is only meant to highlight that there is a possibility that life history traits could also vary in a similar manner to the genetic structure of the two species. We have accordingly changed a few lines in the results to highlight that this is just a preliminary result due to the high uncertainty in sampling, as the reviewer points out, but also suggest that this may be an important avenue for research going forward.

l. 324: *“greater acoustic diversity” Indeed, that can be inferred from the scatterplot, but this is not the same as a greater “vocal differentiation” (l. 367/68). The latter would imply diversified vocal groups, e.g. among mountain systems. At least, in the context of that paragraph it comes across that way, because the previous sentence directly refers to differences among the two species in “populations structure” (M. gigantea does not show a clear [genetic] population structure in contrast to M. lugubris; l. 364). The next sentence says that the vocal pattern is similar, however, although clear genetic clusters have been shown for M. lugburis, this is not true for the songs. Only because the songs are more variable, this does not mean, that this greater variation corresponds to different acoustic entities (dialects for example). At least this has not been shown for songs in the analysis.*

RESPONSE: We thank you for pointing out this statement. Indeed, we should have been more cautious in interpreting the results of the acoustic vocalisations. Due to the low sample size and variation in sampling methods, we could only estimate acoustic variation across the two species. However, our preliminary results indicate that there is more acoustic variation in *M. lugubris* which could potentially relate to different populations and may thus be interesting for future studies. We have included a line in the results which hopefully takes your suggestion into account and highlights the need to look at life history traits in these birds in a more robust manner. We have also refrained from using the term vocal differentiation, as you suggest.

l. 269: *“We recover the same pattern of lower levels of differentiation in M. gigantaea compared to M. lugubris in the PCAs (Fig. 2 A) …” I do not understand this statement. Isn’t the whole paper on the clear differences between the species, e.g. M gigantaea being one very homogenic cluster in the PCA, and M. lugubris being represented by two clear PCA clusters and even a clear clustering structure for k=6. How does this conform with the statement in l. 281 that “Both heterozygosity and nucleotide diversity were significantly lower in M. gigantaea than in M. lugubris”? How can that paragraph be started with the statement that patterns of low (intraspecific) differentiation were the same in the two study species?*

RESPONSE: We apologise for any confusion that may have arisen due to our phrasing. We meant to say that the pattern that we saw in the phylogeny where *M. gigantaea* appears as less diverse than *M. lugubris* is observed in the same way in PCA, admixture and heterozygosity. We have rephrased this sentence and hope it is clearer now.

**Minor**
*In legend of figures 1, 2, 3 4 attention should be paid to the correct italic format of all scientific names.*

RESPONSE: Thank you, we have corrected the legends of all figures to correctly italicise all scientific names.
