# Supplementary material for: Species-specific dynamics may cause deviations from general biogeographical predictions – evidence from a population genomics study of a New Guinean endemic passerine bird family (Melampittidae)
Source: PLoS One. 2024 May 23;19(5):e0293715. doi: 10.1371/journal.pone.0293715 (PMC11115331; doi:10.1371/journal.pone.0293715)
Supplement: Attachment — Submitted filename: ResponseToReviewers.docx [file pone.0293715.s025.docx]

**Journal Requirements:** *Please review your reference list to ensure that it is complete and correct. If you have cited papers that have been retracted, please include the rationale for doing so in the manuscript text, or remove these references and replace them with relevant current references. Any changes to the reference list should be mentioned in the rebuttal letter that accompanies your revised manuscript. If you need to cite a retracted article, indicate the article’s retracted status in the References list and also include a citation and full reference for the retraction notice.*

RESPONSE: We have double checked our reference list for completion and we did not see any citation that has been retracted at this point. Based on the reviewer’s suggestions we have additionally included the following references that were not used in the original manuscript (no reference has been removed):

32.	McCullough JM, Hruska JP, Oliveros CH, Moyle RG, Andersen MJ. Ultraconserved elements support the elevation of a new avian family, Eurocephalidae, the white-crowned shrikes. Ornithology. 2023 Jul 11;140(3):ukad025.

42.	Andrews S. Babraham Bioinformatics - FastQC A Quality Control tool for High Throughput Sequence Data [Internet]. 2010. Available from: <https://www.bioinformatics.babraham.ac.uk/projects/fastqc/>

43.	s4hts/HTStream [Internet]. Software (for) High Throughput Sequencing; 2023 [cited 2024 Jan 15]. Available from: https://github.com/s4hts/HTStream

44.	Bolger AM, Lohse M, Usadel B. Trimmomatic: a flexible trimmer for Illumina sequence data. Bioinformatics. 2014 Aug 1;30(15):2114–20.

45.	Zhang J, Kobert K, Flouri T, Stamatakis A. PEAR: a fast and accurate Illumina Paired-End reAd mergeR. Bioinformatics. 2014 Mar 1;30(5):614–20.

46.	Shen W, Le S, Li Y, Hu F. SeqKit: A Cross-Platform and Ultrafast Toolkit for FASTA/Q File Manipulation. PLOS ONE. 2016 Oct 5;11(10):e0163962.

49.	Danecek P, Bonfield JK, Liddle J, Marshall J, Ohan V, Pollard MO, et al. Twelve years of SAMtools and BCFtools. GigaScience [Internet]. 2021 Feb;10(2). Available from: https://doi.org/10.1093/gigascience/giab008

50.	Okonechnikov K, Conesa A, García-Alcalde F. Qualimap 2: advanced multi-sample quality control for high-throughput sequencing data. Bioinformatics. 2016 Jan 15;

32(2):292–4.

51.	Neukamm J, Peltzer A, Nieselt K. DamageProfiler: fast damage pattern calculation for ancient DNA. Bioinformatics. 2021 Oct 25;37(20):3652–3.

54.	Hahn C, Bachmann L, Chevreux B. Reconstructing mitochondrial genomes directly from genomic next-generation sequencing reads—a baiting and iterative mapping approach. Nucleic Acids Res. 2013 Jul 1;41(13):e129.

58.	Stegemiller MR, Redden RR, Notter DR, Taylor T, Taylor JB, Cockett NE, et al. Using whole genome sequence to compare variant callers and breed differences of US sheep. Front Genet [Internet]. 2023 Jan 4 [cited 2024 Mar 22];13. Available from: <https://www.frontiersin.org/journals/genetics/articles/10.3389/fgene.2022.1060882/full>

60.	Nguyen LT, Schmidt HA, von Haeseler A, Minh BQ. IQ-TREE: A Fast and Effective Stochastic Algorithm for Estimating Maximum-Likelihood Phylogenies. Mol Biol Evol. 2015 Jan 1;32(1):268–74.

61.	Zhang C, Rabiee M, Sayyari E, Mirarab S. ASTRAL-III: polynomial time species tree reconstruction from partially resolved gene trees. BMC Bioinformatics. 2018 May 8;19(6):153.

62.	Rabiee M, Sayyari E, Mirarab S. Multi-allele species reconstruction using ASTRAL. Mol Phylogenet Evol. 2019 Jan 1;130:286–96.

74.	Lerner HRL, Meyer M, James HF, Hofreiter M, Fleischer RC. Multilocus Resolution of Phylogeny and Timescale in the Extant Adaptive Radiation of Hawaiian Honeycreepers. Curr Biol. 2011 Nov;21(21):1838–44.

**Reviewer #1**: *I am satisfied with the sensibly replies to my comments, as well as the corrections made to the manuscript. I have just one remaining request/suggestion, namely to estimate the level of divergence between lineages using the Dxy-estimate. This is crucial to assess the species status. The authors have argued that sample size prohibits this calculation, but actually Dxy is insensitive to sample size (see also one of my replies below). For reference, see also: Roux et al. 2016 Shedding Light on the Grey Zone of Speciation along a Continuum of Genomic Divergence*

RESPONSE: We hope we have addressed all of your suggestions and have now included estimates of D_xy_. See below for our responses to each point.

*A few replies (referring to line numbers of comments of first revision round):*

Line 109-117: *The authors could consider adding this information to the methods section, so the reader better appreciate the efforts that the authors have put in acquiring this dataset.*

RESPONSE: We have added some additional information in the manuscript to explain our low number of modern samples.

Line 126: *same: useful information, why not include in methods section?*

RESPONSE: We now include a sentence in the methods in which we explain our choice of sequencing read length.

Line 142: *Agreed, 20x is sufficient, but on the other hand, which factors could cause a site or region to have a depth of 20 if the mean is 786? It makes such regions look ‘suspicuous’.*

RESPONSE: We agree that such a heavy dip from the average depth of coverage would look odd. Even in the mitochondria, we would expect certain regions to be more difficult to sequence and assemble, such as the control region, but also repetitive regions. Additionally, our chosen method takes a random subset of 5 000 000 reads, which is normally more than sufficient for a mitochondrial assembly, but due to its random nature there may still be a low chance that a certain region is covered by less reads within the subset.

Line 152: *Thanks for the clarification, which even gives me a potential explanation of patterns observed in my own datasets.*

RESPONSE: Happy to help, we have discussed this issue extensively in our group.

Line 155: *Again, a concise summary of these considerations could be added to the methods section, in order for the reader to appreciate the underlying rationale.*

RESPONSE: We have added a short justification to each of the applied variant calling methods.

Line 162: *I am not fully convinced by this answer. Common approaches are not necessarily correct approaches. ML-phylogenetic inferences aims to reconstruct the most likely phylogenetic model, including the parameters u (mutation rate) and t (branch length), given the data. For a concatenated set of independently evolving loci, each with its own u and t, would this mean that in practice the method aims to infer the most likely average values of u and t (?). And if the input dataset is unphased, how does the method deal with ambiguous sites stemming from heterozygosity?*

RESPONSE: The first step of the used workflow (nf-phylo) is to generate a consensus sequence for every chromosome/scaffold based on the vcf of each individual, with heterozygous and low/high coverage positions masked. These consensus sequences are then used as input for phylogenetic inference, not the SNP-data itself. Although we do not reach the point of parameters for each independent locus, we try to accommodate for this issue by inferring phylogenies at different windows sizes (between 2 000 – 20 000 bp) and verify that the topology remains consistent. Additionally, we compare topologies between concatenated and summary coalescent phylogenies to make sure that these are also similar. Another measure that gives us a sense of support from each locus is obtained through the site concordance factor which measures the proportion of sites within each window that support a certain branch.

Ambiguous heterozygous sites are not an issue in our approach as we remove all heterozygous sites during the filtering of our SNP data. We definitely lose some information from these sites by removing them, however it gives us more security due to the relatively low coverage of some of our samples. We have clarified this part in the text (previously it only said “masked”).

Line 178: *Actually, I have to correct here myself. In the original paper, Tajima applies the test both to single-locus and multi-locus datasets.*

RESPONSE: Thank you for the correction. Your original comment was still very much valid, and we have also discussed with other colleagues that it is unusual to present Tajima’s D as a single value for the entire genome as one may miss regions with a strong different signal otherwise. Although we did not intend on investigating the genomes on this scale within this study, it is still important to show changes in Tajima’s D across the entire genome.

Line 295-317: *As a ‘second-opinion’, the authors could calculate Dxy (mean absolute genetic distance), for example using the software PIXY, or using the python scripts from the github page of Simon Martin (distMat.py, or popgenWindows.py –analysis popPairDist). Note that when inputting snp data, you would afterwards have to correct for this by multiplying the output estimates by the proportion of variable sites.*

RESPONSE: We have included estimates of D_xy_ which are now part of the supplementary material (S6 Table). We chose to calculate it through a genotype likelihood-based approach so that we could include as many individuals as possible. The script is based on <https://github.com/mfumagalli/ngsPopGen/blob/master/scripts/calcDxy.R> and was only modified to perform multiple pairwise comparisons and provide a summary of global estimates. The main calculation of D_xy_ remains the same. The approach is described in the methods and codes are listed in S2 File.

Line 399-401: *Unlike Fst, Dxy is not sensitive to sample size. This is one of the main advantages of Dxy over Fst. (Another advantage is that it is not effective by Ne.) Thus, Dxy is even valid in case each population is represented by a single individual only. When using entire genomes, single-locus stochastics are cancelled out by the law or large numbers.*

RESPONSE: Thank you for the comment, although we were sceptical whether D_xy_ would really be representative for single individuals, we were pleasantly surprised to find that pairwise estimates were around the same range even when using different subpopulations (smaller or even consisting of a single individual) for the calculation.
